# Disentangling Polymorphism, Texture, and Molecular Orientation in TIPS-Pentacene Thin Films by Polarized Raman Spectroscopy

**DOI:** 10.1021/acsaom.6c00277

**Published:** 2026-07-06

**Authors:** Martina Zangari, Francesco Stancari, Tommaso Salzillo, Adrian Tamayo, Marta Mas-Torrent, Lorenzo Soprani, Gabriele D’Avino, Francesco Mezzadri, Matteo Masino, Patrizio Graziosi, Elisabetta Venuti

**Affiliations:** † Department of Industrial Chemistry “Toso Montanari” & INSTM-UdR Bologna, 9296 University of Bologna, Via P. Gobetti 85, Bologna 40129, Italy; ‡ Dipartimento di Scienze Chimiche, Della Vita e Della Sostenibilità Ambientale & INSTM-UdR Parma, Parco Area delle Scienze, 17/A, Parma 43124, Italy; § Institut de Ciència de Materials de Barcelona, ICMAB-CSIC, Campus UAB, Bellaterra 08193, Spain; ∥ Dipartimento di Scienze Molecolari e Nanosistemi, Via Torino 155, Venezia Mestre 30172, Italy; ⊥ ISMN−CNR, Consiglio Nazionale delle Ricerche, Via P. Gobetti 101, Bologna 40129, Italy

**Keywords:** TIPS-pentacene, organic semiconductors, polymorphism, polarized Raman spectroscopy, grazing-incidence X-ray diffraction, thin films, molecular orientation, density functional theory

## Abstract

Thin-film processing can alter the crystal structure of organic semiconductors, making it difficult to disentangle the respective roles of polymorphism, texture, and molecular orientation in the charge transport. Here, highly oriented thin films of 6,13-bis­(triisopropylsilylethynyl)­pentacene (TIPS-pentacene) prepared by bar-assisted meniscus shearing are investigated by combining grazing-incidence X-ray diffraction, polarized Raman spectroscopy, and periodic first-principles calculations. We show that bar-assisted meniscus shearing does not generate a distinct thin film polymorph under the investigated conditions. Instead, the films adopt the bulk Form I structure while developing pronounced out-of-plane and in-plane textures. Grazing-incidence X-ray diffraction establishes the film texture, while polarized Raman spectroscopy and periodic density functional theory calculations support the Form I assignment through the angular dependence and low-frequency vibrational fingerprint. In particular, the calculations reproduce the phonon frequencies and polarization dependence, support the vibrational assignment, and show that low-frequency modes in TIPS-pentacene have mixed inter- and intramolecular character, which makes them especially sensitive to subtle packing differences. Comparison among the reported polymorphs further shows that their inherent structure energetics, Raman fingerprints, and transport-related properties remain clearly distinguishable. Overall, this work establishes polarized Raman spectroscopy, combined with grazing-incidence X-ray diffraction and theory, as an effective route to resolve polymorph, texture, and molecular orientation in highly ordered organic semiconductor films.

## Graphical abstract

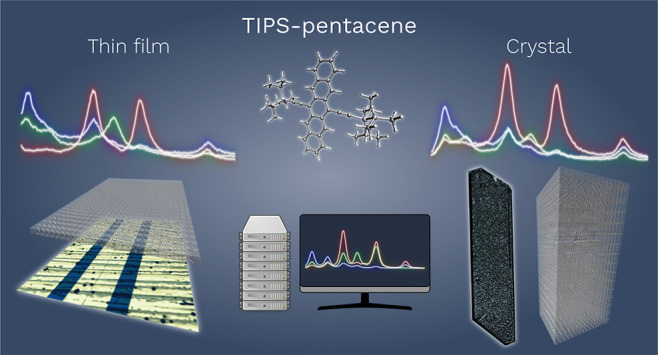

## Introduction

Organic semiconductors (OSCs) are widely used in flexible, low-power devices such as OLEDs, OPVs, and OFETs.^1^ Their performance is governed by charge transport through a π -conjugated molecular solid and is therefore highly sensitive to intermolecular packing.^2,3^ Because transfer integrals, reorganization energies, and dynamic disorder all depend on how molecules arrange in the crystal, identifying and controlling packing motifs are essential for understanding and optimizing charge transport in OSCs. In practical devices, OSCs are most often employed in the form of thin films. However, thin films frequently adopt structures that differ from their thermodynamic bulk phases.^4^ Surface interactions, confinement, and deposition conditions can stabilize polymorphs or metastable arrangements not observed in bulk crystals, directly influencing transfer integrals and OFET mobility.^5^ At the same time, film thickness, degree of crystallinity, and molecular orientation with respect to the substrate can further modulate charge transport, making the relationship between processing and performance particularly complex.^2^ As a result, disentangling the respective roles of polymorphism, texture, and orientational order remains a central challenge in solution-processed OSCs.^6^

Among small-molecule OSCs, pentacene derivatives play a central role. In particular, 6,13-bis­(triisopropylsilylethynyl)­pentacene and derivatives (TIPS-pentacene) have replaced unsubstituted pentacene in many device architectures due to their improved solubility, chemical stability, and high mobility in solution-processed thin films.^7–9^ TIPS-pentacene is also a benchmark system because its charge-transport properties are strongly dependent on molecular packing. Indeed, in situ X-ray scattering has shown that nanoconfinement can direct the formation of distinct thin-film polymorphs with unique π stacking geometries and backbone orientations, including a previously unidentified thin-film-only phase,^10^ with important consequences for charge transport. Beyond thin films, TIPS-pentacene single crystals have been shown to display a reversible shape-memory effect linked to a temperature-induced martensitic transition, in turn triggered by rotation of the bulky side chains.^11^ More recently, mechanical studies have shown that TIPS-pentacene single crystals can undergo stress-induced and reversible polymorphic transitions.^12,13^ Under applied load, the crystal lattice rearranges cooperatively to form a metastable daughter phase, which disappears upon unloading. This reversible transformation gives rise to pronounced superelastic and ferroelastic behavior.

Given the remarkable structural diversity of TIPS-pentacene, ranging from thin-film polymorphs appearing under confinement to thermally or mechanically induced structural transitions in single crystals, a key question is whether highly oriented thin films produced by solution processing reproduce the room-temperature bulk structure or instead access metastable packing motifs. This distinction is not trivial because strong texture can mask or mimic structural differences in diffraction data, while charge-transport properties are influenced simultaneously by polymorph identity and molecular orientation. Even if the room-temperature bulk phase is not necessarily the most favorable from a charge transport perspective, it retains the practical advantage of being the thermodynamically stable form under ambient conditions. Establishing robust structural fingerprints that can discriminate between polymorph and orientational order is therefore essential for rational film design and for building a reliable link between processing, structure, and mobility in this model organic semiconductor.

By combining low-frequency Raman spectroscopy, X-ray diffraction, and solid-state first-principles DFT simulations, we show that thin films produced by bar-assisted meniscus shearing adopt the same crystal structure and molecular arrangement as those of bulk single crystals grown under equilibrium conditions. More specifically, our results indicate that these films can be assigned to bulk Form I while displaying a pronounced orientational texture. This structural invariance persists across a wide range of coating speeds. Such consistency confirms the possibility of polymorph selection in a material often regarded as packing sensitive, suggesting that TIPS-pentacene can be driven toward a single, thermodynamically preferred lattice configuration during film formation. More broadly, this work confirms that polarized low-frequency Raman spectroscopy, when combined with GI-XRD and periodic calculations, provides an effective route to distinguish polymorph, texture, and molecular orientation in highly ordered organic semiconductor thin films.^14,15^

## Materials and Methods

### Crystal Growth

TIPS-pentacene single crystals were grown from a hexane solution by slow solvent evaporation. The crystallization process yielded flat platelet-like crystals with well-defined faces suitable for subsequent characterization.

### Thin Film Transistor Fabrication

TIPS-pentacene films were deposited by bar-assisted meniscus shearing (BAMS) under ambient conditions, following the procedure reported in ref. 16. A 4 wt % solution of TIPS-pentacene in chlorobenzene was deposited onto Si/SiO_2_ substrates (SiO_2_ thickness: 200 nm, C = 17.26 × 10^–9^ F cm^–2^) prepatterned by photolithography with source–drain electrodes consisting of 5 nm Cu and 40 nm Au deposited by thermal evaporation. During BAMS deposition, the hot bed was maintained at 105 °C, and the coating speed was varied between 0.5 and 10 mm s^–1^. Since the coating speed modifies the residence time of the meniscus and therefore the amount of material deposited per unit area, film thickness was controlled indirectly by varying this parameter while keeping all other deposition conditions constant. RMS roughness and thickness as a function of deposition speed were determined by AFM and are reported in the ESI (Table S1 and Figure S1).

### X-ray Diffraction

Single-crystal X-ray diffraction data were collected by using a Bruker D8 Venture diffractometer equipped with a Photon II 2D detector and a Cu microsource. Measurements were performed at 300 and 120 K, and data were refined full matrix with anisotropic displacement parameters for all atoms except hydrogens, refined as riding.

Grazing incidence X-ray diffraction data were collected using a Rigaku SmartLab XE diffractometer with a parallelized CuK_α_ incident beam shaped into a 0.5 mm spot. A HyPix3000 detector was operated in 2D mode to acquire out-of-plane scattering information related to the texture of the sample.

### Raman Spectroscopy

Raman measurements were performed using complementary experimental setups, depending on the spectral range and measurement geometry required. Polarized Raman spectra were acquired in backscattering geometry with a Horiba LabRAM HR Evolution confocal Raman microscope using the 633 nm excitation line. The incident and scattered light were linearly polarized along either the X (horizontal) or Y (vertical) direction of the laboratory reference system. Single crystals were mounted with the crystallographic *b*-axis aligned along the Y direction. The LabRAM HR Evolution setup includes an ultralow-frequency module, allowing access to the low-wavenumber spectral region. Additional Raman spectra were acquired with a confocal Raman microscope (Horiba Xplora Plus, Horiba) equipped with edge filters, enabling measurements down to 50 cm^–1^ from the Rayleigh line. A 785 nm diode laser was used as an excitation source, with reduced power at the sample to minimize thermal damage, and spectra were collected through a 100× objective. Raman spectra of the 10 mm s^–1^ thin films were instead collected with a Jobin Yvon T6400 triple-monochromator spectrometer interfaced with an Olympus BX40 microscope in confocal configuration, using 349 nm excitation from a CW DPSS Skylark laser and a 20× LMU-NUV objective. For each sample, spectra were collected at several points to ensure reproducibility. Data analysis was carried out with Origin 2025.

### DFT

PBE–PAW pseudopotentials^17,18^ and the D3-BJ Grimme scheme with Becke–Johnson damping^19^ were used throughout the DFT calculations performed with VASP^20,21^ to account for van der Waals interactions.^14,22–24^ Calculations started from the experimental unit cell parameters, and the optimal *k*-point grid was determined using a cutoff energy of 400 eV; *k*-point samplings of 7 × 6 × 3 and 5 × 4 × 2 were found to be adequate to achieve energy convergence for polymorphs I and II and polymorph III, respectively. After identifying a converged *k*-grid, the energy cutoff for the wave functions was optimized, reaching convergence at 1000 and 900 eV for polymorphs I and II and polymorph III, respectively, in combination with the optimal k-grids above, with convergence around 0.1 meV/atom. Finally, atomic positions were relaxed while keeping the unit cell parameters fixed at their experimental values, with target convergence criteria of 10^–8^ eV on the energy and 10^–6^ eV/Å on the forces. Following relaxation, vibrational modes and Raman spectra were evaluated using two approaches, which gave consistent results within a tolerance of 0.5 cm^–1^. The Phonopy^25^ package was used to compute and diagonalize the dynamical matrix within the finite-displacements (FD) scheme, using a 2 × 2 × 2 supercell, validated to ensure convergence of phonon frequencies and the 3D density of states (DOS) with all positive real frequencies, except for the acoustic branches at Γ, where a small negative frequency around 10^–1^ to 10^–2^ THz may appear and is considered acceptable. The rigid-molecule character of the modes was evaluated by projecting the eigenvectors onto rotational and translational coordinates in the molecular inertia-axes system, using the symmetry.py code.^26^ Finally, the off-resonant Raman activities were computed with the vasp_Raman.py program.^27^ This program uses VASP as backend to compute the polarizability with the finite displacement approach and returned the Raman activity of the selected modes. Vibrational properties and Raman intensities have also been computed with the PhonoMol code adopting a complete basis of displacements (no minimal molecular displacements approximation),^28^ enabling the direct evaluation of the composition of each mode in terms of rigid-molecule translations and rotations and intramolecular vibrations. Throughout the text, we use *k* and *q* to indicate the BZ points and the corresponding wave vectors of electrons and phonons, respectively.

For the polymorphs I and II, mobility values were also computed following the approach described in ref. 15,23 , based on the evaluation of the electron–phonon coupling as modulation of the transfer integrals from the bandwidth over the BZ and including both this modulation and the phonon population in the solution of the Boltzmann transport equation. Additionally, the scattering induced by a phenomenological barrier was included, following the approach outlined in ref. 15.

## Results and Discussion

### Single-Crystal Structure Description

TIPS-pentacene is known to exhibit three bulk polymorphs, conventionally denoted Forms I, II, and III, reported in the Cambridge Structural Database (CSD) under the refcodes VOQBIM and VOQBIM01–02,^7,11,29^ VOQBIM03,^11^ and VOQBIM04,^13^ respectively. Form I is the polymorph stable at ambient conditions, whereas Forms II and III are accessed upon heating at 397 and 444 K, respectively.^13^ Structural studies show that the three forms retain the same general packing motif but differ in unit–cell parameters and, most notably, in the degree of conformational order of the triisopropyl side chains.^7,11,13,29^ Establishing the polymorph adopted by the reference single crystals is therefore important for comparison with thin films and for the interpretation of the Raman response.

In this work, single-crystal X-ray diffraction measurements were performed at room temperature and 120 K. Within this temperature range, the examined crystals were found to be consistent with Form I, i.e., the ambient-stable polymorph used here as the bulk structural reference. The crystals adopted platelet-like or needle-like habits, with the longest dimension oriented parallel to the crystallographic *b*-axis in direct space, while the *c*-axis was approximately perpendicular to the largest crystal face. The crystallographic orientation of the examined single crystals is shown in Figure 1, while the corresponding cell parameters are listed in Table 1. Additional details on structure refinement and agreement factors are provided in the ESI, Table S2.

**Figure 1:**
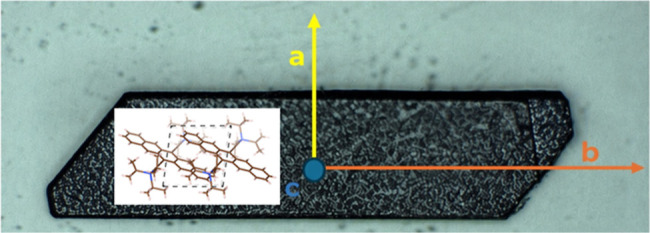
Optical image of a TIPS-pentacene single crystal used as the structural reference, with the crystallographic axes superimposed. The crystal elongation direction is parallel to the *b*-axis, while the *a*-axis lies in the plane of the major face; the *c*-axis is approximately perpendicular to the crystal surface. The inset shows the molecular arrangement of Form I viewed along a direction consistent with the crystal face, with the dashed box indicating the projected unit cell.

**Table 1:** Unit-Cell Parameters Obtained for TIPS-Pentacene Form I from Single-Crystal X-ray Diffraction Measurements Collected at Room Temperature and at 120 K, Compared with Literature Values Reported at 173 K^7^

	*a* (Å)	*b* (Å)	*c* (Å)	α (◦)	β (◦)	γ (◦)
RT	7.7430(17)	7.7571(17)	16.944(4)	88.479(9)	77.791(9)	82.161(9)
120 K	7.5403(7)	7.7348(7)	16.7904(16)	89.476(4)	78.531(4)	83.792(4)
^7^(173 K)	7.5650(15)	7.7500(15)	16.835(3)	89.15(3)	78.42(3)	83.63(3)

No significant structural changes were observed between room temperature and 120 K, indicating the absence of any temperature-induced phase transitions within this interval. As expected, the unit–cell parameters refined at a low temperature show improved agreement with the literature values reported at 173 K by Anthony et al. in ref. 7. Overall, the examined crystals are consistent with the previously reported Form I phase, and no evidence was found for additional phases or structural modifications under the present measurement conditions.

### Thin Film Structure Description

Grazing-incidence X-ray diffraction (GI-XRD) measurements were performed on the TIPS-pentacene films using a 2D detector to probe a wide region of reciprocal space. The films exhibited well-defined diffraction features, confirming their good crystalline quality. Distinct diffraction spots were observed at low angles, while the ring detected at 2θ ≈ 38.2° was attributed to the gold contacts (ICSD # 01–089–3697). As a representative example, the diffraction pattern of the sample deposited at a coating speed of 0.5 mm/s is shown in Figure 2.

**Figure 2:**
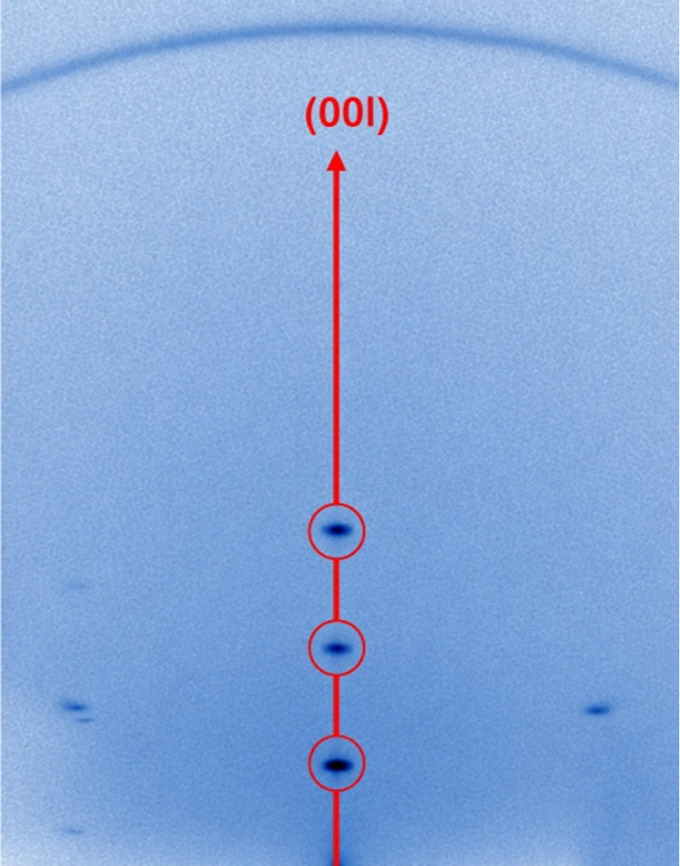
Grazing-incidence XRD pattern of a TIPS-pentacene thin film prepared by the BAMS deposition technique using a coating speed of 0.5 mm/s.

The diffraction features observed along the surface normal direction could be indexed as 00l reflections, indicating a strong out-of-plane preferential orientation. This texture is consistent with a molecular arrangement in which the π-conjugated pentacene cores are oriented largely upright with respect to the substrate, as illustrated in Figure 3.

**Figure 3:**
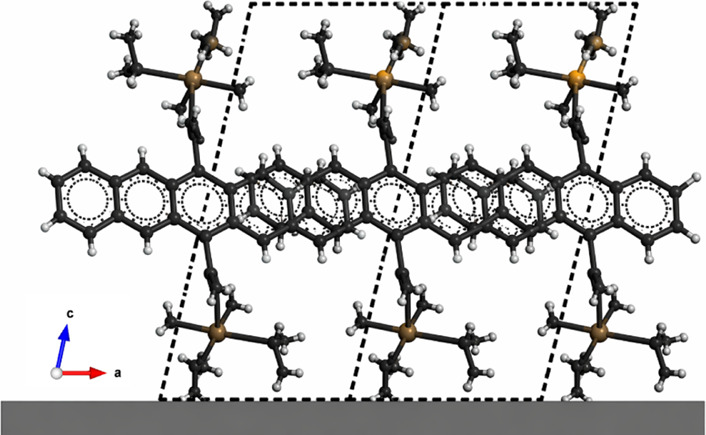
Illustration of the molecular packing adopted by TIPS-pentacene in the thin film, highlighting the preferential 00l orientation, with the π-conjugated pentacene cores oriented largely upright with respect to the substrate. The dashed black lines indicate the projection of the primitive unit cell in the plane of the drawing, while the gray horizontal bar represents the substrate. The crystallographic a and c axes are indicated in the lower-left corner.

Additional information on the in-plane texture was obtained from the diffraction spots located away from the surface normal. Notably, rotation of the sample around the 00l axis (φ angle) produced a clear modulation of the diffraction pattern, demonstrating the presence of a pronounced in-plane texture rather than the typical fiber-like orientation expected for non-epitaxial films, where rotation around the surface normal would yield an invariant pattern (Figure 4a).

**Figure 4:**
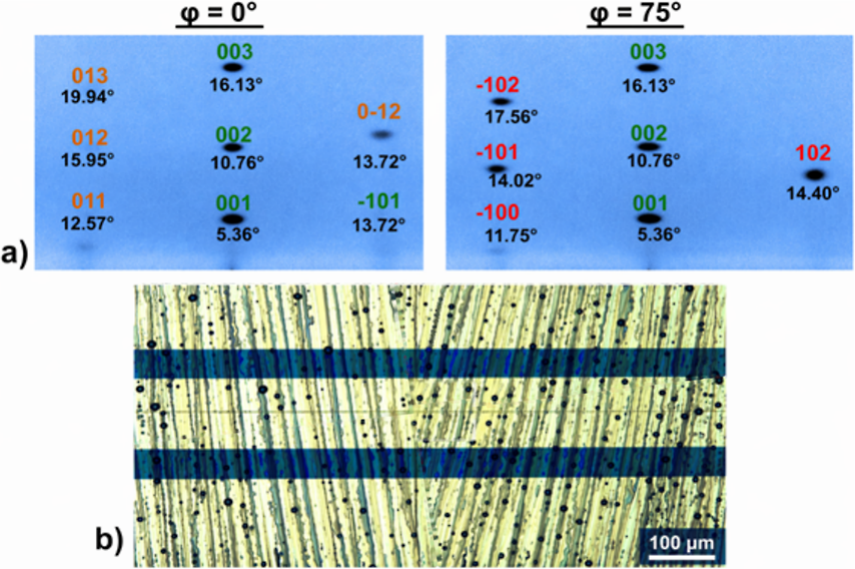
(a) Evolution of the GI-XRD pattern upon rotation of the sample around the 00l axis (φ angle). The φ-dependent modulation of the diffraction spots indicates a well-defined in-plane texture, showing that the film is not fiber-textured but exhibits preferential in-plane crystallographic alignment. (b) Reflected-light optical microscope image of the TIPS-pentacene film surface on the prepatterned device substrate. The dark horizontal bands correspond to the Cu/Au source–drain electrodes. The striped domains are associated with the strong in-plane crystallographic texture of the film.

The comparison of the experimental reciprocal-space maps with those calculated from the single-crystal XRD structure allowed the observed diffraction spots to be indexed on the basis of both *d*-spacing and reciprocal-space position, supporting the assignment of the film structure to Form I. The analysis also showed that the area illuminated by the incident beam (0.5 × 0.5 mm^2^) was dominated by a single crystallographic orientation. Upon translating the sample under the beam, different diffraction patterns were obtained, indicating the presence of multiple domains with slightly different in-plane crystallographic orientations (Figure 4a). Representative GI-XRD patterns collected at different sample positions are reported in Figure S2. The present GI-XRD measurements were not intended as a spatially resolved domain-size mapping; therefore, a quantitative distribution of domain sizes cannot be extracted from these data. However, the changes observed upon translation are consistent with the stripe-like morphology seen by optical microscopy and with the presence of preferentially oriented domains on the scale probed by the X-ray beam. Thus, although the film was not a single crystal, it displayed a pronounced in-plane texture, in agreement with the striped morphology observed by optical microscopy (Figure 4b).

The GI-XRD analysis thus supports the assignment of the films to Form I and demonstrates their strong texture. To further corroborate this assignment and obtain direct information about molecular orientation, we used polarized Raman spectroscopy as a complementary vibrational probe of both angular response and low-frequency lattice fingerprints.

### Raman Spectra Analysis

Raman spectra of TIPS-pentacene were collected on both bulk crystals and thin films, covering low- and high-frequency regions in order to assess the possible presence of different polymorphs and, through polarization-dependent measurements of both lattice and intramolecular modes, to probe the molecular orientation and alignment of the films with respect to the substrate.

As a first step, polarized Raman measurements were used to determine the orientation of the molecular frame with respect to the crystal morphology in both single-crystal and thin film samples. The single crystal was taken as a structural reference since its morphology and crystallographic orientation could be independently established by X-ray diffraction. TIPS-pentacene bulk crystals grow as elongated plates, with the most developed face corresponding to the ab plane, while the long crystal direction coincides with the crystallographic *b*-axis (Figure 1). This provides a well-defined crystallographic framework for the interpretation of the polarization-dependent Raman response.

To identify the orientation of the molecule within this frame, angle-dependent polarized Raman spectra were collected in parallel polarization geometry by rotating the sample in the plane of the ab face. In the adopted convention, an angle of 0° corresponds to the crystallographic *b*-axis, i.e., to the elongation direction of the plate-like crystal. The angular series measured on the single crystal (Figure 5, left panel) shows a pronounced modulation of the overall Raman intensity, with a clear maximum at 150° and a minimum approximately 90° apart, at 60°. This behavior directly reflects the anisotropy of the Raman molecular polarizability and provides information on the orientation of the molecular axes within the crystal.

**Figure 5:**
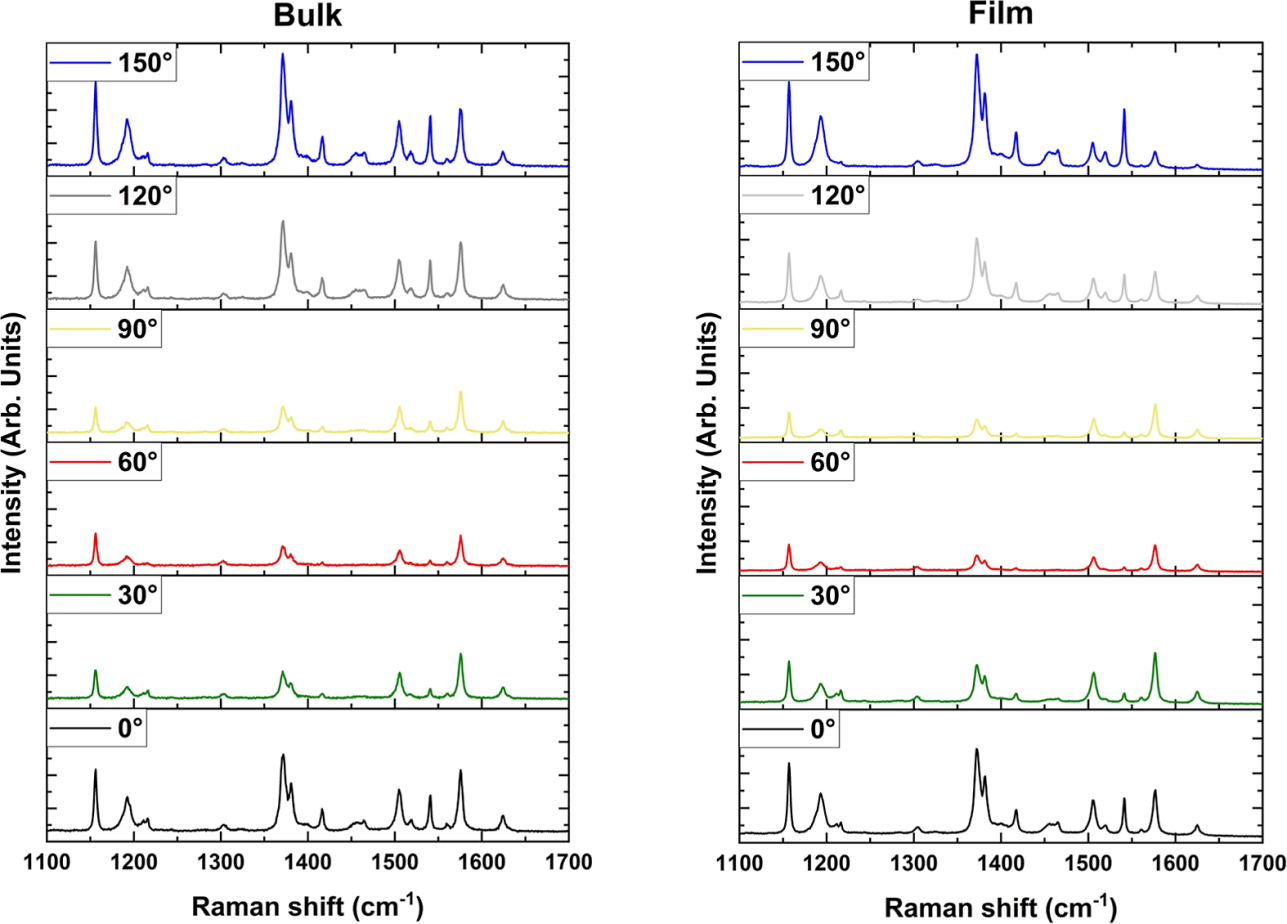
Polarized high-frequency Raman spectra acquired for the bulk crystal (left) and the thin film (right). The comparison shows that the maximum and minimum signal intensities are obtained at the same polarization angles for both samples.

The strongest Raman response is expected when the electric field of the incident light is aligned with the direction associated with the largest variation of molecular polarizability. In TIPS-pentacene, this direction is naturally identified with the long axis of the conjugated pentacene backbone.^30,31^ Accordingly, the intensity maximum observed at (150°) indicates that the long axis of the pentacene backbone is oriented approximately 150° with respect to the crystallographic *b*-axis. This assignment is fully consistent with the molecular arrangement obtained from the X-ray structure and relies on the angular dependence of the Raman polarizability response. In addition, we note that the use of 785 nm excitation may introduce pre-resonant contributions to the Raman intensities which are not considered in the calculations.

The same analysis was then extended to the thin-film samples. In this case, the reference direction for the angular scan (0° angle) was chosen along the direction parallel to the stripe-like domains observed by optical microscopy (see Figure 4b). As shown in Figure 5 (right panel), the thin film exhibits the same angular dependence as the single crystal, with the Raman intensity reaching a maximum for the same polarization direction (150°). This result demonstrates that the thin film retains a clear in-plane orientational order and indicates that the stripe direction corresponds to the same crystallographic *b*-axis. Although the film is not a single crystal, its polarized Raman response reveals the presence of preferentially oriented crystalline domains sharing a common in-plane alignment.

This comparison between the bulk crystal and thin film represents the first main result of the Raman analysis. The single crystal provides the structural benchmark required to assign the molecular orientation, while the corresponding angular dependence observed in the film demonstrates that the BAMS layers preserve a significant degree of orientational order in the growth plane. Angle-dependent polarized Raman spectroscopy therefore proves to be an effective and non-destructive tool for probing molecular alignment in TIPS-pentacene thin films and establishes the experimental basis for the symmetry-based interpretation of the polarized Raman spectra discussed below. Thus, polarized Raman provides two complementary pieces of information: the angular high-frequency response identifies the in-plane molecular orientation, whereas the low-frequency lattice modes identify the polymorph.

Low-frequency Raman spectra collected on the bulk single crystal and on the thin film deposited at a low deposition speed (0.5 mm/s) exhibit the same polarized patterns and relative intensity distribution, with no additional bands observed in the thin film (Figure 6, left panel). Since low-frequency Raman modes are highly sensitive to intermolecular packing, the absence of additional features in the film indicates that the same polymorph is preserved upon BAMS deposition, in agreement with the X-ray diffraction results. Raman spectra of films deposited at different coating speeds, reported in the ESI (Figure S3), further confirm that the deposited phase is always Form I.

**Figure 6:**
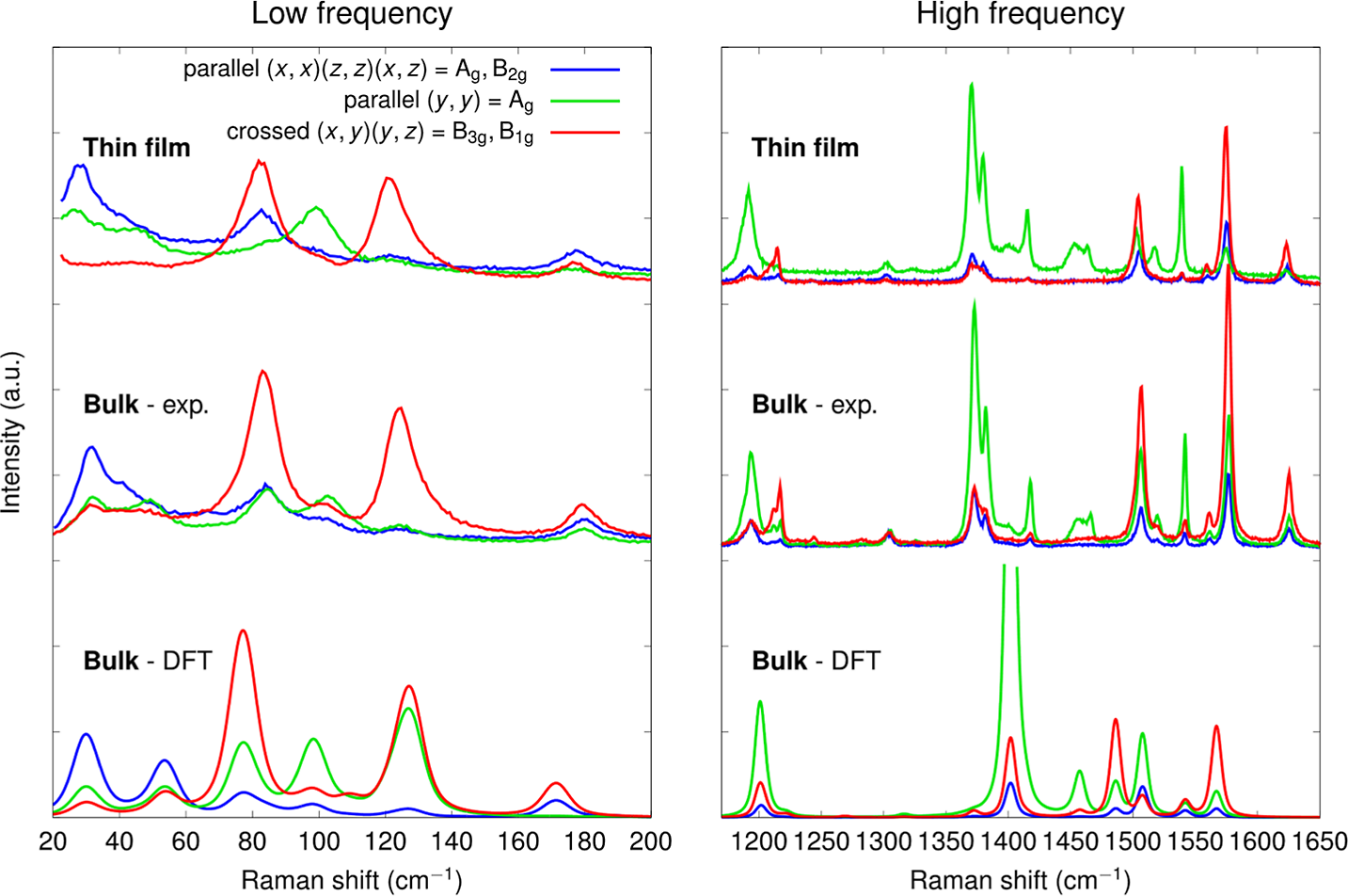
Polarized Raman spectra of TIPS-pentacene acquired at room temperature on the bulk single crystal and on the thin film deposited by BAMS at a coating speed of 0.5 mm s^–1^ in selected ranges. Spectra are shown for the three selected polarization configurations, (150°,150°), (60°,60°), and (60°,150°), defined in the molecular reference frame discussed in the text together with the corresponding DFT simulations (intensities calculated in the 20–200 and 1150–1600 cm^–1^ spectral ranges). The bulk crystal and thin film display the same set of low-frequency bands and polarization dependence.

Having established that no evidence of polymorphism emerges from the comparison between the bulk crystal and thin films, the polarized spectra can be analyzed in terms of mode symmetry. A symmetry analysis performed strictly in the crystallographic frame is, however, of limited usefulness for TIPS-pentacene. The crystal is triclinic P1̅, with Z = 1, and all Raman-active modes formally have Ag symmetry; as a result, measurements along crystallographic axes alone do not provide an efficient classification of the observed vibrations. We therefore adopt an oriented-gas model and express the Raman response in the molecular frame, which provides a more effective classification of the observed vibrations. This approach is justified by the presence of a single molecule per unit cell, which implies the absence of Davydov splitting and allows the crystal polarizability to be related to the molecular polarizability tensor together with the molecular orientation in the unit cell. Within this framework, the vibrational modes can be approximately classified by exploiting the pseudo-*D*_2h_ symmetry of the TIPS-pentacene molecule. Here, pseudo-*D*_2h_ indicates that, although the system is not characterized by *D*_2h_ symmetry, the molecule, and in particular the pentacene backbone, can be described within an approximate *D*_2h_-like symmetry framework.

The molecular reference frame is defined with (Y) along the long molecular axis of the pentacene backbone, (X) normal to the pentacene skeleton, and (Z) approximately along the direction joining the two silicon atoms (see Figure S4 of the ESI). Once the orientation of the molecular frame has been established from the angle-dependent polarized Raman measurements discussed above, the experimental polarization geometries can be translated to specific components of the molecular Raman tensor.

In this convention, the (150°,150°) configuration corresponds predominantly to the (Y,Y) component and therefore selectively enhances totally symmetric A_g_ modes of the *D*_2h_ symmetry group. The (60°,60°) configuration is more complex because the molecule is tilted with respect to the ab plane; accordingly, both totally symmetric contributions associated with (X,X) and (Z,Z) and non-totally symmetric contributions of B_2g_-type associated with (X,Z) can contribute to this polarization. Finally, the crossed (60°,150°) configuration contains mainly (X,Y) and (Z,Y) tensor components, thereby activating modes of B_1g_ and B_3g_ symmetry.

This polarization scheme is particularly useful in the low-frequency region, where collective intermolecular vibrations dominate the Raman response. As shown in Figure 6, the spectra collected in the three selected polarization configurations, (150°,150°), (60°,60°), and (60°,150°), display marked differences in the relative intensities of the bands. In particular, two intense features at about 81 and 122 cm^–1^ are selectively activated in crossed polarization and are absent or strongly suppressed in the parallel spectra. Their behavior identifies them as non-totally symmetric lattice modes with pronounced librational character.

The same symmetry-based approach can be extended to the high-frequency region, where the Raman spectrum is dominated by intramolecular vibrations of the pentacene core and TIPS substituents. As shown in Figure 6 (right panel), the polarized spectra retain the same selection pattern observed in the lattice-mode region, allowing the bands to be classified within the same pseudo-*D*_2h_ framework. In particular, characteristic bands of the pentacene backbone in the 1620, 1575, and 1505 cm^–1^ intervals are selectively activated in crossed polarization, consistent with their non-totally symmetric character and with the expected response of the conjugated core. The fact that the same polarization behavior is observed in both the single crystal and thin film further supports the conclusion that the film preserves not only the same crystal phase but also the same average molecular arrangement within the growth plane.

## DFT Calculations

### Raman Spectra Simulations

Periodic DFT calculations were used to validate the polarization-dependent Raman assignment, compare the low-frequency fingerprints of the known polymorphs, and connect the structural assignment to charge-transport-relevant lattice dynamics.

As shown in Figure 6, the calculated spectra reproduce the main features of the polarized low-frequency Raman pattern, both in terms of peak positions and polarization dependence. Projection of the calculated Raman tensors onto the experimental polarization directions also accounts for the observed selection rules and relative enhancement/suppression of the main bands, supporting the reliability of the calculated frequencies and of the associated mode assignments. Some deviations in relative intensities are expected because Raman intensities depend on the absolute values and orientation of the Raman-tensor components and are therefore sensitive to experimental factors such as optical alignment, finite polarization extinction, domain orientation/averaging, focusing and collection conditions, and resonance/pre-resonance effects not included in the off-resonant harmonic calculations. A list of the calculated frequencies in the spectral range of interest is reported in Table S3. The calculations are especially informative in the low-frequency region, where the observed bands originate from lattice phonons. Analysis of the calculated eigenvectors identifies those vibrations with the strongest lattice contribution and helps clarify their symmetry assignment within the pseudo-*D*_2h_ molecular framework. In particular, the experimental features observed at about 81 and 122 cm^–1^, selectively activated in crossed polarization, are reproduced by calculated modes in the same spectral region and are found to have a pronounced non-totally symmetric librational character. Likewise, the intense low-energy band observed around 30 cm^–1^ in the (60°,60°) configuration is well matched by the lowest-frequency calculated mode, whose eigenvector is dominated by intermolecular motion. These results also indicate that, although the low-frequency modes retain a strong lattice character, they are not purely intermolecular and instead display a partial admixture of intramolecular motion, as expected for a relatively large and flexible molecular crystal. The simulated polarization dependence further confirms that the (150°,150°) geometry is dominated by totally symmetric contributions, whereas the crossed (60°,150°) configuration selectively enhances non-totally symmetric modes. This result is fully consistent with the experimental selection pattern and supports the oriented-gas model as an effective, although approximate, description of the Raman response of TIPS-pentacene.

A consistent correspondence is also obtained in the high-frequency region, although the large density of intramolecular modes makes the comparison more sensitive to frequency offsets and relative intensity differences. As shown in Figure 6, the calculated intramolecular modes reproduce the main experimental peak positions and their polarization dependences. As additional support, the comparison between the experimental angular dependence and the corresponding calculated Raman response in the high-frequency region is reported in the ESI, Figure S5. The calculated band around 1400 cm^–1^ arises from a group of closely spaced intramolecular modes of TIPS-pentacene. Some components have low calculated Raman activity and are weak on the plotted scale; moreover, unscaled harmonic periodic DFT frequencies are not expected to give an exact one-to-one match in this high-frequency region. Notably, the characteristic features of the pentacene core in the 1500–1600 cm^–1^ spectral range are accurately reproduced and display the expected enhancement in crossed polarization, consistent with their non-totally symmetric character within the pseudo-*D*_2h_ scheme. Overall, the comparison between experiment and periodic DFT calculations provides a coherent microscopic interpretation of the vibrational properties of TIPS-pentacene, linking the observed polarization dependence to specific vibrational motions and symmetry features of the molecule in the crystal.

### Polymorphism in TIPS-Pentacene

The agreement between the experiment and periodic DFT discussed in the previous section already indicates that the investigated films display the same vibrational fingerprint as the bulk Form I crystal. To place this assignment on a broader basis, the relevant TIPS-pentacene polymorphs are compared below in terms of inherent structure energetics, calculated vibrational fingerprints, and charge-transport properties. This broader comparison is important because polymorph identification in this system is not only a structural issue but also directly affects both the Raman response and the expected transport behavior.

### Inherent Structures

To assess whether the reported polymorphs correspond to genuinely distinct mechanically stable configurations, we adopted an inherent-structure approach, namely, the full relaxation of the experimental crystal structures to their nearest local minima on the potential-energy surface.^32,33^ Full unconstrained relaxations were therefore carried out starting from the three reported crystal structures, VOQBIM01 (Form I), VOQBIM03 (Form II), and VOQBIM04 (Form III). For all three polymorphs, a 5 × 4 × 2 *k*-point grid and a wave function cutoff energy of 900 eV were used, and the structures were relaxed until the forces were below 1 × 10^–5^ eV/Å. The resulting energies are summarized in Table 2. In all cases, the relaxation leads to a distinct local minimum, showing that Forms I, II, and III represent genuine polymorphic structures. Their energy ranking provides the energetic framework for the discussion below and can be compared with the experimentally observed sequence of polymorph stability. Interestingly, although the dispersive contribution is slightly more stabilizing for Form II than for Form I, the total energy ordering is determined by the larger electronic stabilization of Form I, which emerges as the lowest-energy structure among the three polymorphs.

**Table 2:** Calculated Total Energies of the Relaxed Inherent Structures of TIPS-Pentacene Forms I, II, and III, Together with Their Decomposition into Electronic and Dispersive Contributions

energy in eV	Form I	Form II	Form III
total energy	–573.47	–573.41	–573.23
electronic contribution	–564.60	–564.40	–564.34
dispersive contribution	–8.87	–9.01	–8.88

### Calculated vibrational Fingerprints of the TIPS-Pentacene Polymorphs

As noted above for Form I, Table S3 of the ESI provides the complete list of calculated low-frequency modes for Forms I, II, and III, including symmetry labels, Raman activity, and their decomposition into rigid-molecule rotational contributions along the three principal inertia axes. The comparison shows that the three polymorphs display distinct vibrational fingerprints, although some similarities remain, particularly between Forms I and II. In particular, the mode decomposition reported in Table S3 indicates that, despite partial overlap in frequency, both the relative Raman activities and the rotational character of the lattice modes remain clearly polymorph-dependent. Because of these partial similarities, especially between Forms I and II, a mode-by-mode comparison alone may not always be sufficient for an unambiguous polymorph assignment. For this reason, the Raman response was also considered to be at the level of the full spectral profile. Powder (unpolarized) Raman spectra were therefore computed in the lattice phonon region for the relevant polymorphs. As shown in Figure S6 of the ESI, the overall spectral patterns remain clearly polymorph-dependent, despite the partial overlap of some individual modes. The calculated spectra thus confirm that the experimental Raman response is fully consistent with Form I and does not support the presence of alternative polymorphs in the investigated specimens.

### Charge Transport Properties

Mobility calculations were then carried out for polymorphs I and II and compared with the single-crystal field-effect transistor (FET) data reported in ref. 34. The experimental values measured at gate voltages closest to the band-like regime were selected and are reported in Figure 7 in black. Thus, for Form I, the mobility along the average direction of the *a* and *b* crystallographic axes was first computed by including electron–phonon scattering together with scattering from ionized point defects, assuming a defect density equal to the carrier density;^23^ the corresponding results are shown in green. A second set of calculations were then performed by combining electron–phonon scattering with scattering due to an effective energy barrier, in the absence of ionized impurities;^15^ these results are shown in yellow. Such an effective barrier can be regarded as a phenomenological way to account for additional microstructural barriers not explicitly included in the model, such as grain- or twin-boundary effects reported in TIPS-pentacene films.^35^ In the former case, the defect density that maps the mobility at 300 K was considered, whereas in the latter, the same procedure was applied to the barrier height. An effective barrier of 110 meV was found to reproduce both the absolute mobility and its temperature dependence. For Form II, by contrast, the same analysis requires a substantially higher barrier, namely, 240 meV (magenta), because the phonon-limited mobility is intrinsically higher, and its temperature dependence differs more markedly from the experimental behavior. This comparison therefore indicates that Form I provides the more realistic description of the measured transport response.

**Figure 7:**
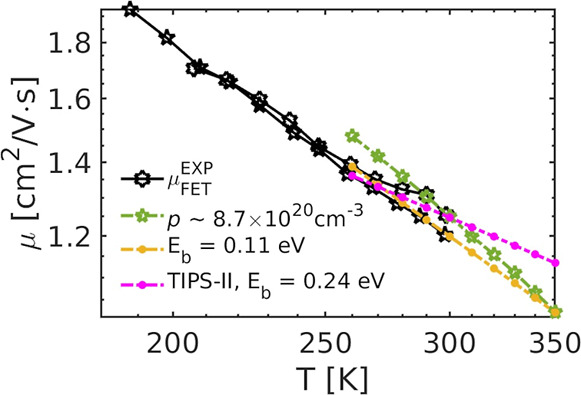
Comparison between the experimental single-crystal field-effect transistor (FET) mobility from ref. 34 and the computed mobility for TIPS-pentacene polymorph I under different scattering approximations. In addition to electron–phonon scattering, scattering either from ionized point defects (green) or from an effective energy barrier (yellow) is included. For polymorph II, only the effective-barrier case is reported. Experimental mobility values are reproduced from ref. 34 since no error bars or numerical uncertainties are provided in the original report, the data are shown here without uncertainty estimates. The calculated curves correspond to deterministic model outputs under the stated scattering assumptions.

The in-plane mobility anisotropy in the ab plane was then evaluated under the two disorder models described above and is reported in Figure 8. The left-hand panel refers to Form I, whereas the right-hand panel refers to Form II. The corresponding average mobility values and anisotropy ratios are reported in the legend. Form II is characterized not only by generally higher mobility values but also by a markedly lower anisotropy than Form I. This behavior appears less consistent with the investigated specimens and, therefore, provides further support for the assignment of the experimental samples to Form I.

**Figure 8:**
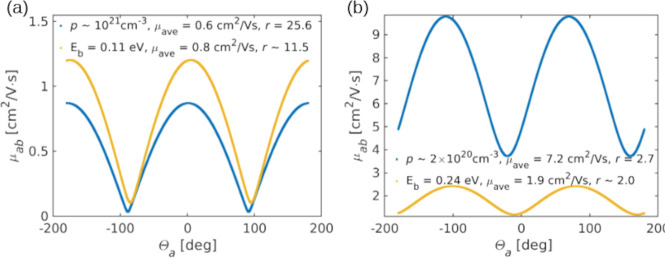
In-plane mobility anisotropy in the ab plane for TIPS-pentacene polymorph I (a) and polymorph II (b), computed by including electron–phonon scattering together with either ionized point defects (blue) or an effective energy barrier (yellow). The relevant values of defect density (p) or barrier (E_b_), average mobility value (m_ave_), and anisotropy mobility ratio (r) are reported in the legend.

A complementary mode-/*q-*point-resolved analysis of the low-energy vibrational contributions to the electron–phonon coupling is reported in Table S4 of the ESI, where the mode/q-point pairs featuring the largest effective EPC are listed. Here, the effective EPC is quantified through the ratio between the EPC and the phonon frequency, so as to account not only for the coupling strength itself but also for the phonon population. The analysis of these modes shows that the largest effective contributions are associated both with low-energy modes having substantial lattice character and with intramolecular modes. This analysis is intended to map the q-resolved EPC landscape, including contributions from zone boundaries beyond Γ. The comprehensive EPC, obtained as a mode-resolved DOS-weighted average over the sampled Brillouin-zone points,^15^ is also reported. We observe that the intermolecular character is mainly dominated by translations along the axis of the pentacene backbone or along the one connecting the Si atoms, consistent with the picture proposed for TIPS-pentacene, in which low-energy structural fluctuations strongly modulate intermolecular electronic coupling and play a key role in charge transport.^36^ However, internal modes may also contribute to the modulation of the electronic coupling and thus affect charge transport.

## Conclusions

By combining GI-XRD, polarized Raman spectroscopy, and periodic first-principles calculations, we show that TIPS-pentacene thin films prepared by bar-assisted meniscus shearing adopt the bulk Form I structure under the investigated conditions while maintaining strong out-of-plane order and a well-defined in-plane texture. Polarized Raman measurements further establish a direct link between the stripe-like film morphology and the crystallographic *b*-axis, providing direct information on the molecular orientation in the contact plane. The agreement between experiment and theory gives a consistent microscopic interpretation of the polarized Raman response and supports the assignment of both low- and high-frequency vibrational modes. In particular, the low-frequency region proves especially sensitive to subtle packing differences in the presence of the mixed lattice–intramolecular character of the vibrational modes in this energy range. The calculations also place the structural assignment on a broader basis. Inherent-structure analysis shows that the literature-reported polymorphs of TIPS-pentacene correspond to genuinely distinct local minima on the potential-energy surface, but remain clearly distinguishable in terms of Raman fingerprints and transport-related properties. Taken together, these results make it possible to discard the alternative polymorphs as viable structural descriptions of the investigated films and identify Form I as both the thermodynamically preferred and the experimentally relevant structure under the present conditions. Mobility calculations based on electron–phonon coupling further reinforce this picture, showing that the measured transport behavior is better described by Form I than by the alternative polymorphs considered here.

Overall, this work establishes polarized Raman spectroscopy, especially when combined with GI-XRD, first-principles calculations, and transport modeling, as an effective and non-destructive route to disentangle polymorphism, texture, and molecular orientation in highly ordered organic semiconductor films. The broader significance is not only the assignment of BAMS TIPS-pentacene films to Form I but also the demonstration that polarized low-frequency Raman spectroscopy can separate polymorph identity from orientational texture, providing a coherent framework for connecting processing, crystal structure, vibrational fingerprints, and charge-transport behavior in solution-processed molecular semiconductors.

## Supplementary Material



## Notes

Film deposition and characterization of TIPS-pentacene thin films; crystallographic data for TIPS-pentacene Form I; supplementary Raman spectra as a function of coating speed; definition of the molecular reference frame for polarized Raman analysis; comparison between experimental and calculated angular dependence of the Raman response; calculated low-frequency Raman fingerprints of TIPS-pentacene polymorphs; and mode-resolved analysis of low-energy electron–phonon coupling and translational/rotational phonon character (PDF)

## References

[1] Sawatzki-Park M., Wang S.-J., Kleemann H., Leo K. (2023). Highly Ordered Small
Molecule Organic Semiconductor Thin-Films Enabling Complex, High-Performance
Multi-Junction Devices. Chem. Rev..

[2] McHugh C. (2024). Crystal Clear:
The Impact of Crystal Structure in the Development of High-Performance
Organic Semiconductors. Acta Crystallogr. Sect.
B:Struct. Sci..

[3] Chen X., Guo J., Peng L., Wang Q., Jiang S., Li Y. (2023). Charge Transport
in Organic Field-Effect Transistors. Mater.
Today Electron..

[4] Shioya N., Gasser F., Strasser N., Zojer E., Resel R., Simbrunner J., Hasegawa T. (2026). From Monolayer to Bulk: Thin-Film-Specific
Polymorphic Transitions of a Molecular Semiconductor. Chem. Mater..

[5] Marina S., Dyson M., Rodríguez-Martínez X., Reid O. G., Li R., Rumbles G., Smilgies D., Amassian A., Campoy-Quiles M., Stingelin N., Martín J. (2024). Using Spatial Confinement to Decipher Polymorphism
in the Organic Semiconductor P-DTS­(FBTTh2)­2. J. Mater. Chem. C.

[6] Salzillo T., Giunchi A., Pandolfi L., Brillante A., Venuti E. (2021). Bulk and Surface-Mediated Polymorphs of Bio-Inspired
Dyes Organic Semiconductors: The Role of Lattice Phonons in Their
Investigation. Isr. J. Chem..

[7] Anthony J. E., Brooks J. S., Eaton D. L., Parkin S. R. (2001). Functionalized Pentacene:
Improved Electronic Properties from Control of Solid-State Order. J. Am. Chem. Soc..

[8] Thorley K. J., Rupasinghe G. S., Ruhunage S., Ogbaje M., Bhat V., Parkin S., Risko C., Paterson A., Anthony J. E. (2026). Simple
Synthesis, Structural and Electronic Characterization of Soluble Contorted
Pentacenes. Chem. Mater..

[9] Freudenberg J., Bunz U. H. F. (2024). How to Stabilize
Large Soluble (Hetero-)­Acenes. J. Am. Chem.
Soc..

[10] Diao Y., Lenn K. M., Lee W.-Y., Blood-Forsythe M. A., Xu J., Mao Y., Kim Y., Reinspach J. A., Park S., Aspuru-Guzik A., Xue G., Clancy P., Bao Z., Mannsfeld S. C. B. (2014). Understanding Polymorphism in Organic
Semiconductor Thin Films through Nanoconfinement. J. Am. Chem. Soc..

[11] Chung H., Dudenko D., Zhang F., D’Avino G., Ruzié C., Richard A., Schweicher G., Cornil J., Beljonne D., Geerts Y., Diao Y. (2018). Rotator Side
Chains Trigger Cooperative Transition for Shape and Function Memory
Effect in Organic Semiconductors. Nat. Commun..

[12] Park S. K., Sun H., Bernhardt M., Hwang K., Anthony J. E., Zhao K., Diao Y. (2023). Unraveling
Molecular Design Principle of Ferroelasticity in Organic
Semiconductor Crystals with Two-Dimensional Brickwork Packing. Chem. Mater..

[13] Park S. K., Sun H., Chung H., Patel B. B., Zhang F., Davies D. W., Woods T. J., Zhao K., Diao Y. (2020). Super- and Ferroelastic
Organic Semiconductors for Ultraflexible Single-Crystal Electronics. Angew. Chem..

[14] Bedoya-Martínez N., Schrode B., Jones A. O. F., Salzillo T., Ruzié C., Demitri N., Geerts Y. H., Venuti E., Della Valle R. G., Zojer E., Resel R. (2017). DFT-Assisted Polymorph Identification
from Lattice Raman Fingerprinting. J. Phys.
Chem. Lett..

[15] Gnoli L., Venuti E., Salzillo T., Masino M., Graziosi P. (2025). Electrons
and Phonons in Pentacene: Coupling Patterns Reveal the Microscopic
Origin of the Phonon Limited Mobility. J. Phys.
Chem. C.

[16] Temiño I., Del Pozo F. G., Ajayakumar M. R., Galindo S., Puigdollers J., Mas-Torrent M. (2016). Mas-Torrent, M. A Rapid, Low-Cost, and Scalable Technique
for Printing State-of-the-Art Organic Field-Effect Transistors. Adv. Mater. Technol..

[17] Kresse G., Joubert D. (1999). From Ultrasoft Pseudopotentials to the Projector Augmented-Wave
Method. Phys. Rev. B:Condens. Matter.

[18] Perdew J. P., Burke K., Ernzerhof M. (1996). Generalized
Gradient Approximation
Made Simple. Phys. Rev. Lett..

[19] Grimme S., Ehrlich S., Goerigk L. (2011). Effect of
the Damping Function in
Dispersion Corrected Density Functional Theory. J. Comput. Chem..

[20] Kresse G., Furthmüller J. (1996). Efficient Iterative Schemes for Ab Initio Total-Energy
Calculations Using a Plane-Wave Basis Set. Phys.
Rev. B:Condens. Matter.

[21] Kresse G., Hafner J. (1993). Ab Initio Molecular Dynamics for
Liquid Metals. Phys. Rev. B:Condens. Matter.

[22] Giunchi A., Pandolfi L., Della Valle R. G., Salzillo T., Venuti E., Girlando A. (2023). Lattice Dynamics of
Quinacridone Polymorphs: A Combined
Raman and Computational Approach. Cryst. Growth
Des..

[23] Graziosi P., Della Valle R. G., Salzillo T., D’Agostino S., Zangari M., Cané E., Masino M., Venuti E. (2025). Electron-Phonon
Coupling and Mobility Modeling in Organic Semiconductors: Method and
Application to Two Tetracene Polymorphs. Phys.
Rev. Mater..

[24] Kamencek T., Wieser S., Kojima H., Bedoya-Martínez N., Dürholt J. P., Schmid R., Zojer E. (2020). Evaluating Computational
Shortcuts in Supercell-Based Phonon Calculations of Molecular Crystals:
The Instructive Case of Naphthalene. J. Chem.
Theory Comput..

[25] Togo A. (2023). First-Principles
Phonon Calculations with Phonopy and Phono3py. J. Phys. Soc. Jpn..

[26] Della
Valle R. G., Halonen L., Venuti E. (1999). Molecular Anharmonicity:
A Computer-Aided Treatment. J. Comput. Chem..

[27] Fonari, A. ; Stauffer, S. Vasp_raman.Py Https://Github.Com/Raman-Sc/VASP/.

[28] Soprani L., Giunchi A., Bardini M., Meier Q. N., D’Avino G. (2025). Accurate and
Efficient Phonon Calculations in Molecular Crystals via Minimal Molecular
Displacements. J. Chem. Theory Comput..

[29] Xu X., Xiao T., Gu X., Yang X., Kershaw S. V., Zhao N., Xu J., Miao Q. (2015). Solution-Processed
Ambipolar Organic Thin-Film Transistors by Blending p- and n-Type
Semiconductors: Solid Solution versus Microphase Separation. ACS Appl. Mater. Interfaces.

[30] Xu J., Diao Y., Zhou D., Mao Y., Giri G., Chen W., Liu N., Mannsfeld S. C. B., Xue G., Bao Z. (2014). Probing the Interfacial Molecular
Packing in TIPS-Pentacene Organic Semiconductors by Surface Enhanced
Raman Scattering. J. Mater. Chem. C.

[31] James D. T., Kjellander B. K. C., Smaal W. T. T., Gelinck G. H., Combe C., McCulloch I., Wilson R., Burroughes J. H., Bradley D. D. C., Kim J.-S. (2011). Thin-Film
Morphology of Inkjet-Printed
Single-Droplet Organic Transistors Using Polarized Raman Spectroscopy:
Effect of Blending TIPS-Pentacene with Insulating Polymer. ACS Nano.

[32] Stillinger, F. H. Interaction Potentials and Inherent Structures in Liquids, Glasses, and Crystals. 16th Annu. Int. Conf. Cent. Nonlinear Stud. 1997, 107(2), 383–391.

[33] Della
Valle R. G., Venuti E., Brillante A., Girlando A. (2003). Inherent Structures of Crystalline Pentacene. J. Chem. Phys..

[34] Sakanoue T., Sirringhaus H. (2010). Band-like Temperature Dependence of Mobility in a Solution-Processed
Organic Semiconductor. Nat. Mater..

[35] Li Y., Wan J., Smilgies D.-M., Miller R., Headrick R. L. (2020). Enhancement of Charge
Transfer in Thermally-Expanded and Strain-Stabilized TIPS-Pentacene
Thin Films. Phys. Rev. Research.

[36] Eggeman A. S., Illig S., Troisi A., Sirringhaus H., Midgley P. A. (2013). Measurement of Molecular Motion in
Organic Semiconductors
by Thermal Diffuse Electron Scattering. Nat.
Mater..

