# Supplementary material for: Disentangling Polymorphism, Texture, and Molecular Orientation in TIPS-Pentacene Thin Films by Polarized Raman Spectroscopy
Source: ACS Appl Opt Mater. 2026 Jul 6;4(7):2224–34. doi: 10.1021/acsaom.6c00277 (PMC13435869; doi:10.1021/acsaom.6c00277)
Supplement: Supplementary file 1 [file ot6c00277_si_001.pdf]

Supporting Information

Disentangling polymorphism, texture, and molecular orientation in TIPS-pentacene thin films by polarized Raman spectroscopy

Martina Zangari,[a†] Francesco Stancari,[b†] Tommaso Salzillo,[a] Adrian Tamayo,[c] Marta Mas-Torrent,[c] Lorenzo Soprani,[a] Gabriele D'Avino[d] Francesco Mezzadri,[b*] Matteo Masino,[b*] Patrizio Graziosi[e], Elisabetta Venuti[a*]*

[a]Department of Industrial Chemistry "Toso Montanari" & INSTM-UdR Bologna, University of Bologna, Via P. Gobetti 85, 40129, Bologna, Italy.

[b]Dipartimento di Scienze Chimiche, Della Vita e Della Sostenibilità Ambientale & INSTM-UdR Parma, Parco Area delle Scienze, 17/A, 43124 Parma, Italy.

[c]Institut de Ciència de Materials de Barcelona, ICMAB-CSIC, Campus UAB, Bellaterra, 08193 Spain.

[d]Dipartimento di Scienze Molecolari e Nanosistemi, Via Torino 155, 30172, Venezia Mestre, Italy.

[e]ISMN – CNR, Consiglio Nazionale delle Ricerche, Via P. Gobetti 101, 40129, Bologna, Italy

† These authors contributed equally to this work and share first authorship

Corresponding authors:

Matteo Masino, matteo.masino@unipr.it

Francesco Mezzadri, francesco.mezzadri@unipr.it

Tommaso Salzillo, tommaso.salzillo@unibo.it

Elisabetta Venuti, elisabetta.venuti@unibo.it

Table of Contents

S1. Film deposition and AFM data

Table S1. RMS roughness and thickness measured by AFM for TIPS-pentacene films as a function of deposition speed. Figure S1. AFM topography images of TIPS-pentacene thin films deposited by BAMS

S2. Crystallographic data

Table S2. Crystal data and details of measurements for crystalline TIPS-pentacene Form I structure. Figure S2. Position-dependent GI-XRD patterns.

S3. Supplementary Raman spectra of films deposited at different coating speeds

Figure S3. Raman spectra of TIPS-pentacene films deposited at different coating speeds.

S4. Molecular reference frame used for polarized Raman analysis

Figure S4. Molecular geometry of TIPS-pentacene with the molecular reference axes used for the polarized Raman analysis.

S5. Experimental and calculated angular dependence of the high-frequency Raman response

Figure S5. Experimental and calculated angular dependence of the high-frequency Raman response of TIPS-pentacene in the (ab) plane of the single crystal.

S6. Calculated low-frequency Raman fingerprints of TIPS-pentacene polymorphs

Figure S6. Calculated low frequency Raman spectra of the reported TIPS-pentacene polymorphs.

Table S3. Calculated and experimental wavenumbers of the Raman-active A_g modes of TIPS-pentacene Forms I, II, and III, together with the calculated squared rotational components around the principal inertia axes.

S7. Mode-resolved analysis of low-energy electron–phonon coupling

Table S4. Optical modes below 100 cm^{-1} together with EPC and EPC/$\hbar\omega$ values.

S1. Film deposition and AFM data

TIPS-pentacene films were deposited by bar-assisted meniscus shearing (BAMS) from a 4 wt% solution in chlorobenzene. The hotbed temperature was maintained at 105 °C, and the coating speed was varied between 0.5 and 10 mm s^{-1}. Surface topographies and film thicknesses of the thin films were examined by atomic force microscopy using a 5500LS Scanning Probe Microscopy system from Agilent Technologies, operated in dynamic mode under ambient conditions. Data analysis was performed using Gwyddion 2.56 software. RMS roughness values were extracted from AFM topographic images after background correction, while film thicknesses were determined from height profiles across film step edges.

*Table S1. RMS roughness and thickness measured by AFM for TIPS-pentacene films as a function of deposition speed.**

Coating speed (mm s^{-1})	RMS roughness (nm)	Thickness (nm)
1	87.4 ± 20.7	292 ± 77
4	21.4 ± 12.1	101 ± 40
10	24.6 ± 7.8	56 ± 12

**The 0.5 mm s^{-1} film was not quantitatively characterized by AFM within this series. Based on the measured thickness trend for the neat films, a thicker film of about 400 nm can be estimated. No reliable estimate of the corresponding roughness can be made because this quantity does not vary monotonically with deposition speed.*

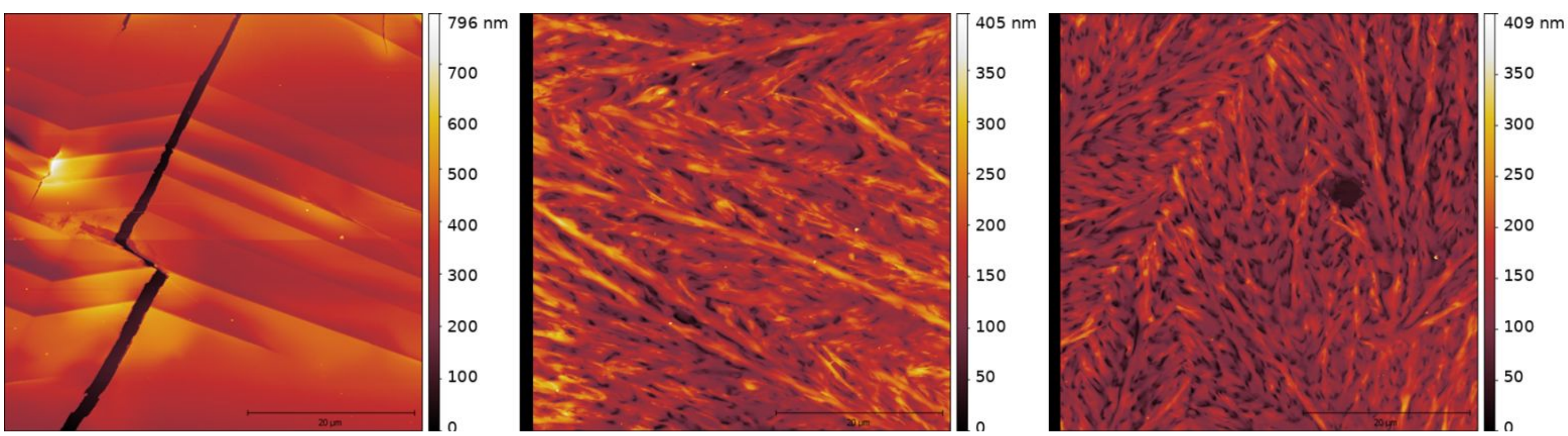

Figure S1. *AFM topography images of TIPS-pentacene thin films deposited by BAMS from a 4 wt% chlorobenzene solution at a hotbed temperature of 105 °C. The coating speeds are, from left to right,*

1, 4, and 10 mm s^{-1}. The color scales indicate height, and the horizontal scale bar is 20 µm. These measurements were used to evaluate the RMS roughness and film thickness reported in Table S1.

S2. Crystallographic data

The single-crystal X-ray diffraction measurements reported in the main text confirm that the examined crystals are consistent with Form I between room temperature and 120 K. Final refinement statistics and additional crystallographic details are given in Table S2.

Table S2. Crystal data and details of measurements for crystalline TIPS-Pentacene Form I

300K

Space Group P-1<br>a=7.7430(17)Å, b=7.7571(17)Å, c=16.944(4)Å, α=88.479(9)°, 77.791(9)°, γ=82.161(9)°, V=985.4(4) Å^3<br>GooF = S = 1.033, R_1 = 0.0906 for 2100 Fo > 4σ(F_o) and 0.1967 for all 40255 data<br>214 parameters, 0 restraints										
atom	x	y	z	Uiso	U11	U22	U33	U23	U13	U12
Si01	0.24595(14)	0.78964(14)	0.17840(6)	0.0707(4)	0.0810(7)	0.0697(8)	0.0608(7)	0.0188(5)	-0.0111(5)	-0.0179(5)
C1	0.5763(9)	0.5974(17)	0.1823(6)	0.305(8)	0.113(5)	0.420(18)	0.298(12)	0.239(13)	0.055(6)	0.059(7)
H1A	0.640392	0.627195	0.22151	0.457						
H1B	0.658414	0.538721	0.13752	0.457						
H1C	0.49107	0.522192	0.206476	0.457						
C002	0.1247(4)	0.3929(4)	0.53739(19)	0.0568(8)	0.078(2)	0.0433(19)	0.0530(18)	0.0069(14)	-0.0171(16)	-0.0170(16)
C003	0.0551(4)	0.5620(4)	0.41992(19)	0.0569(8)	0.074(2)	0.0422(18)	0.0584(19)	0.0057(14)	-0.0165(16)	-0.0171(16)
C004	0.4269(5)	0.2487(4)	0.5325(2)	0.0631(9)	0.078(2)	0.047(2)	0.069(2)	-0.0005(16)	-0.0231(18)	-0.0115(17)
C005	0.1795(4)	0.4545(4)	0.45651(19)	0.0575(8)	0.076(2)	0.0431(18)	0.0556(19)	0.0054(14)	-0.0148(16)	-0.0145(15)
C006	0.2528(5)	0.2897(4)	0.5732(2)	0.0630(9)	0.085(2)	0.046(2)	0.064(2)	0.0099(15)	-0.0251(18)	-0.0157(17)
H006	0.218575	0.248703	0.625491	0.076						
C007	0.4797(5)	0.3082(4)	0.4519(2)	0.0633(9)	0.076(2)	0.047(2)	0.069(2)	-0.0027(16)	-0.0175(18)	-0.0123(17)
C008	0.1118(4)	0.6291(4)	0.3409(2)	0.0619(9)	0.075(2)	0.055(2)	0.057(2)	0.0073(16)	-0.0144(16)	-0.0133(16)

C009	0.3569(5)	0.4091(4)	0.4162(2)	0.0633(9)	0.077(2)	0.053(2)	0.060(2)	0.0048(16)	-0.0119(17)	-0.0169(17)
H009	0.392979	0.448222	0.363668	0.076						
C00A	0.5587(5)	0.1445(5)	0.5683(2)	0.0726(10)	0.091(3)	0.055(2)	0.076(2)	0.0064(18)	-0.029(2)	-0.010(2)
H00A	0.526885	0.10311	0.620613	0.087						
C00B	0.1616(5)	0.6900(5)	0.2754(2)	0.0685(9)	0.077(2)	0.065(2)	0.062(2)	0.0128(18)	-0.0106(17)	-0.0118(18)
C00C	0.7262(5)	0.1069(5)	0.5269(3)	0.0818(11)	0.082(3)	0.070(3)	0.099(3)	0.000(2)	-0.037(2)	-0.004(2)
H00C	0.809678	0.039946	0.551156	0.098						
C00D	0.6621(5)	0.2640(5)	0.4110(2)	0.0755(10)	0.078(2)	0.066(3)	0.082(3)	0.001(2)	-0.011(2)	-0.017(2)
H00D	0.699729	0.303402	0.358634	0.091						
C00E	0.7789(6)	0.1664(6)	0.4476(3)	0.0855(12)	0.081(3)	0.075(3)	0.102(3)	-0.006(2)	-0.023(2)	-0.007(2)
H00E	0.89635	0.137698	0.420045	0.103						
C00F	0.1417(6)	0.6983(6)	0.1017(3)	0.0941(13)	0.120(3)	0.090(3)	0.077(3)	0.015(2)	-0.023(2)	-0.029(3)
H00F	0.013392	0.732156	0.121475	0.113						
C00G	0.1752(7)	1.0284(6)	0.1871(3)	0.0967(14)	0.142(4)	0.067(3)	0.082(3)	0.020(2)	-0.025(3)	-0.018(3)
H00G	0.221263	1.080848	0.134959	0.116						
C00H	0.4932(7)	0.7389(10)	0.1568(4)	0.148(3)	0.082(3)	0.180(6)	0.170(6)	0.107(5)	-0.014(3)	-0.016(3)
C00I	-0.0295(9)	1.0703(8)	0.2053(4)	0.145(2)	0.168(6)	0.105(5)	0.137(5)	0.017(4)	-0.012(4)	0.034(4)
H00B	-0.07429	1.034309	0.160553	0.217						
H00H	-0.06313	1.193271	0.213629	0.217						
H00I	-0.07877	1.009394	0.253047	0.217						
C00J	0.1799(9)	0.7788(8)	0.0198(3)	0.131(2)	0.198(6)	0.137(5)	0.079(3)	0.027(3)	-0.054(3)	-0.060(4)
H00J	0.148441	0.902703	0.024429	0.197						

H00K	0.111188	0.733341	-0.01385	0.197						
H00L	0.304533	0.751943	-0.00389	0.197						
C00K	0.1630(11)	0.5045(8)	0.0981(4)	0.161(3)	0.275(8)	0.104(5)	0.136(5)	0.015(4)	-0.095(5)	-0.067(5)
H00M	0.287401	0.460075	0.083135	0.241						
H00N	0.102074	0.469094	0.058712	0.241						
H00O	0.113294	0.45968	0.150055	0.241						
C00L	0.2520(10)	1.1120(8)	0.2524(4)	0.152(2)	0.257(8)	0.089(4)	0.125(5)	0.008(3)	-0.063(5)	-0.045(5)
H00P	0.224595	1.050175	0.302316	0.228						
H00Q	0.19988	1.23155	0.260151	0.228						
H00R	0.379015	1.106011	0.234963	0.228						
C00M	0.5870(7)	0.8393(11)	0.0887(4)	0.165(3)	0.089(3)	0.231(8)	0.168(6)	0.090(6)	-0.009(3)	-0.039(4)
H00S	0.565888	0.800285	0.038713	0.248						
H00T	0.712665	0.821597	0.087483	0.248						
H00U	0.542849	0.96089	0.096034	0.248						

All s.o.f.s =1

120K

Space Group P-1										
a=7.5403(7)Å, b=7.7348(7)Å, c=16.7904(16)Å, α=89.476(4)°, β=78.531(4)°, γ=83.792(4)°, V= 954.00(15)$Å^3$										
GooF = S = 1.041, R_1 = 0.0741 for 3458 $F_o > 4\sigma(F_o)$ and 0.0995 for all 4719 data										
214 parameters, 0 restraints										
atom	x	y	z	Uiso	U11	U22	U33	U23	U13	U12
Si01	0.24599(8)	0.20919(9)	0.82303(4)	0.0259(2)	0.0228(3)	0.0272(4)	0.0278(4)	0.0097(3)	-0.0046(2)	-0.0052(2)
C002	0.3893(3)	0.3705(3)	0.65990(15)	0.0247(5)	0.0224(10)	0.0219(11)	0.0306(13)	0.0037(9)	-0.0069(9)	-0.0026(8)
C003	0.4466(3)	0.4387(3)	0.58025(14)	0.0226(5)	0.0246(10)	0.0190(11)	0.0252(12)	0.0039(9)	-0.0056(8)	-0.0056(8)
C004	0.6273(3)	0.3928(3)	0.53714(14)	0.0222(5)	0.0247(11)	0.0176(10)	0.0257(12)	0.0031(9)	-0.0070(9)	-0.0045(8)

C005	0.0130(3)	0.6939(3)	0.55195(14)	0.0243(5)	0.0238(11)	0.0207(11)	0.0292(12)	-0.0003(9)	-0.0062(9)	-0.0036(9)
C006	0.7577(3)	0.2908(3)	0.57233(14)	0.0237(5)	0.0260(11)	0.0195(11)	0.0271(12)	0.0040(9)	-0.0079(9)	-0.0036(8)
H006	0.723516	0.250809	0.626288	0.028						
C007	0.9343(3)	0.2477(3)	0.53022(14)	0.0236(5)	0.0257(11)	0.0173(11)	0.0294(12)	0.0015(9)	-0.0089(9)	-0.0031(8)
C008	0.1396(3)	0.5926(3)	0.58693(14)	0.0241(5)	0.0247(11)	0.0211(11)	0.0266(12)	0.0036(9)	-0.0044(9)	-0.0046(9)
H008	0.104081	0.553658	0.641	0.029						
C009	0.3192(3)	0.5457(3)	0.54476(14)	0.0224(5)	0.0233(10)	0.0174(10)	0.0273(12)	0.0030(9)	-0.0065(9)	-0.0033(8)
C00A	-0.0687(3)	0.8545(3)	0.43450(16)	0.0278(5)	0.0295(12)	0.0219(12)	0.0340(13)	0.0034(10)	-0.0115(10)	-0.0029(9)
H00A	-0.03715	0.893849	0.380276	0.033						
C00B	-0.2397(3)	0.8956(3)	0.47744(16)	0.0306(5)	0.0278(12)	0.0267(13)	0.0394(15)	0.0019(10)	-0.0135(10)	-0.0003(9)
H00B	-0.32654	0.963638	0.453005	0.037						
C00C	0.3363(3)	0.3091(3)	0.72579(15)	0.0280(5)	0.0242(11)	0.0294(13)	0.0307(13)	0.0070(10)	-0.0063(9)	-0.0036(9)
C00D	-0.1695(3)	0.7406(3)	0.59480(16)	0.0281(5)	0.0248(11)	0.0263(12)	0.0322(13)	0.0002(10)	-0.0034(9)	-0.0026(9)
H00D	-0.20574	0.702995	0.649054	0.034						
C00E	0.3550(3)	0.3057(3)	0.90168(15)	0.0309(5)	0.0324(12)	0.0315(13)	0.0314(13)	0.0074(10)	-0.0098(10)	-0.0092(10)
H00E	0.489442	0.283391	0.881322	0.037						
C00F	0.3172(4)	-0.0325(3)	0.81330(16)	0.0334(6)	0.0436(14)	0.0272(13)	0.0302(14)	0.0084(10)	-0.0087(11)	-0.0056(11)
H00F	0.26782	-0.08596	0.866442	0.04						
C00G	-0.2915(3)	0.8381(3)	0.55879(16)	0.0311(6)	0.0234(11)	0.0278(13)	0.0411(15)	-0.0024(11)	-0.0060(10)	0.0007(9)
H00G	-0.41227	0.868259	0.588041	0.037						
C00H	0.3161(4)	0.2199(4)	0.98493(18)	0.0445(7)	0.0550(18)	0.0500(18)	0.0359(16)	0.0120(13)	-0.0187(13)	-0.0213(14)
H00C	0.392632	0.262791	1.019605	0.067						
H00H	0.34322	0.093478	0.97825	0.067						
H00I	0.187585	0.248547	1.010247	0.067						
C00I	0.2389(5)	-0.1177(4)	0.74752(19)	0.0484(8)	0.070(2)	0.0343(16)	0.0445(18)	0.0023(13)	-0.0158(15)	-0.0126(15)

H00J	0.28259	-0.24195	0.743289	0.073						
H00K	0.278595	-0.06242	0.695203	0.073						
H00L	0.105673	-0.10313	0.762108	0.073						
C00J	0.5249(4)	-0.0726(4)	0.7967(2)	0.0455(7)	0.0478(17)	0.0386(16)	0.0462(18)	0.0042(13)	-0.0071(13)	0.0091(13)
H00M	0.5575	-0.19867	0.791686	0.068						
H00N	0.572277	-0.02746	0.841711	0.068						
H00O	0.577572	-0.01718	0.746042	0.068						
C00K	-0.1046(4)	0.1545(5)	0.91321(19)	0.0463(8)	0.0244(12)	0.061(2)	0.0527(18)	0.0225(15)	-0.0032(12)	-0.0108(12)
H00P	-0.23675	0.17855	0.918553	0.069						
H00Q	-0.06969	0.190062	0.963305	0.069						
H00R	-0.0685	0.029678	0.903589	0.069						
C00L	-0.0096(4)	0.2555(5)	0.8421(2)	0.0529(9)	0.0232(13)	0.064(2)	0.071(2)	0.0397(18)	-0.0071(13)	-0.0076(13)
H00S	-0.03177	0.181962	0.797143	0.063						
C00M	0.3113(6)	0.5038(4)	0.9107(2)	0.0577(9)	0.088(3)	0.0346(17)	0.059(2)	0.0024(15)	-0.0341(19)	-0.0118(17)
H00T	0.385718	0.548379	0.945949	0.087						
H00U	0.182135	0.532355	0.9349	0.087						
H00V	0.338289	0.557212	0.857139	0.087						
N	-0.0928(6)	0.4057(12)	0.8221(5)	0.212(6)	0.039(2)	0.286(10)	0.261(9)	0.229(8)	0.045(4)	0.050(4)
HA	-0.19224	0.382696	0.795262	0.318						
HB	-0.00527	0.468498	0.784963	0.318						
HC	-0.14218	0.476282	0.871235	0.318						

All s.o.f.s =1

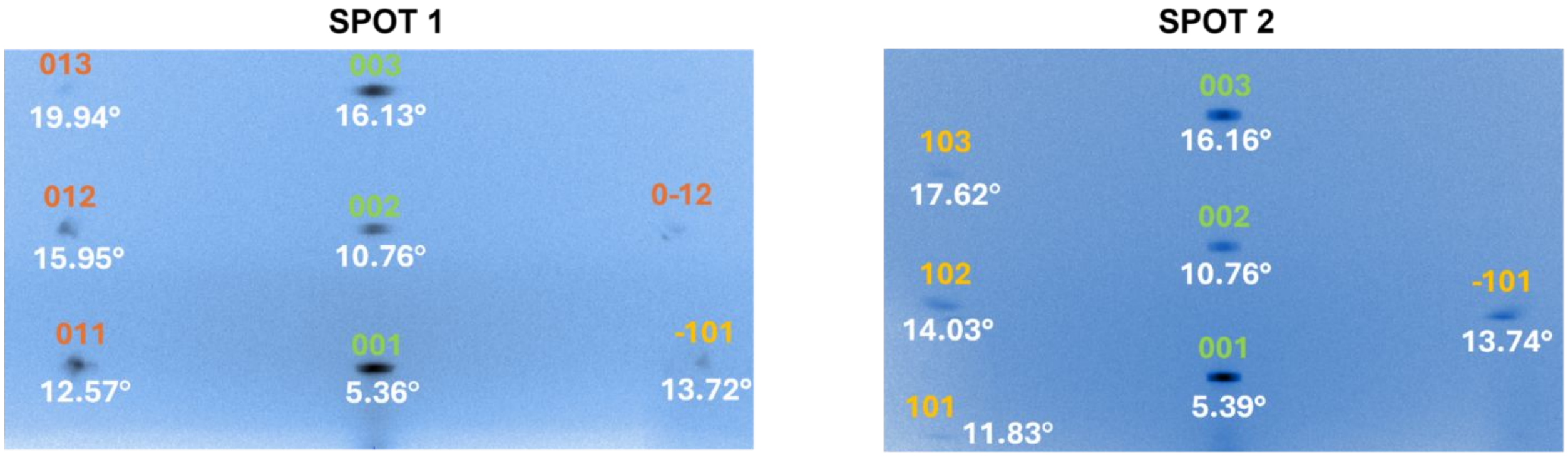

Figure S2. GI-XRD patterns acquired at two different positions of the sample, showing domains with the same 00l out-of-plane orientation and different in-plane orientations. Crystallographic indices in orange and yellow correspond to different domains, whereas green labels indicate reflections common to all orientations.

S3. Supplementary Raman spectra of films deposited at different coating speeds

Raman spectra collected on films deposited at different coating speeds provide a direct check of phase preservation across the BAMS series. The comparison reported below shows that the low frequency spectral pattern remains consistent with Form I throughout the investigated conditions, with no evidence of additional polymorph-specific features. The phase assignment is based on the persistence of the Form I band positions and overall vibrational fingerprint. Differences in relative band intensities are attributed to the strong orientation dependence of the Raman response in these textured films, together with local variations in domain orientation, film thickness, focusing/collection conditions, and, for the 10 mm s^{-1} sample, the different excitation wavelength.

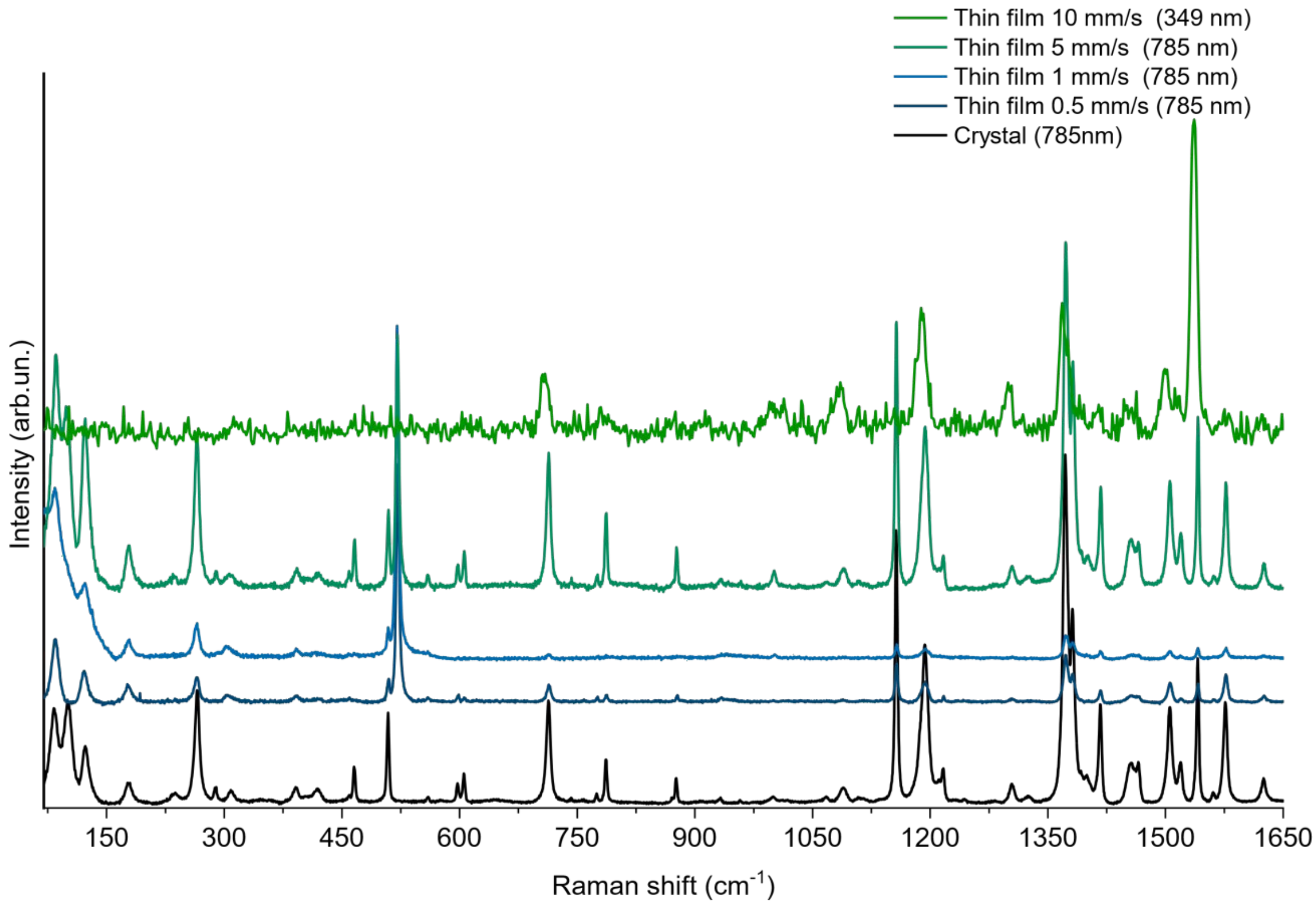

Figure S3. Raman spectra of TIPS-pentacene films deposited at different coating speeds in the 50-1650 cm^{-1} wavenumber range. The spectra were acquired using 785 nm excitation, with the exception of the 10 mm/s film, for which a 349 nm excitation was used to compensate for the weak scattering expected from its lower thickness and to exploit resonance enhancement. The spectra confirm preservation of the Form I vibrational fingerprint in the series fabricated in the investigated BAMS conditions.

S4. Molecular reference frame used for polarized Raman analysis

The oriented gas description adopted in the main text is expressed in the molecular frame. In this convention, Y is taken along the long axis of the pentacene backbone, X is normal to the pentacene skeleton, and Z lies along the Si···Si direction. This frame is used to interpret the polarization selection rules discussed for both the low- and high-frequency Raman spectra.

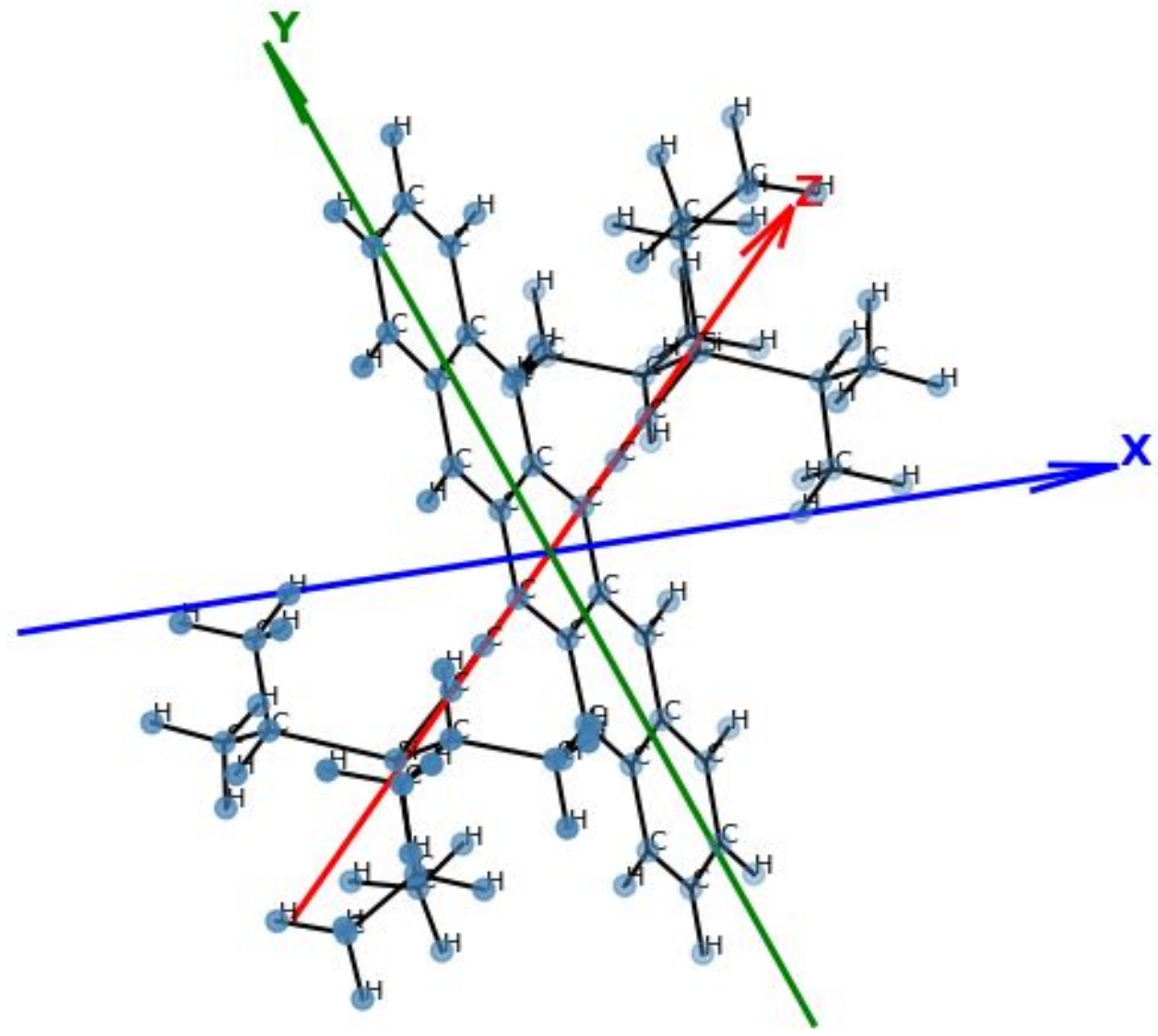

Figure S4. Molecular geometry of TIPS-pentacene with the molecular reference axes used for the polarized Raman analysis. The X, Y, and Z axes define an orthogonal molecular reference frame, corresponding to the principal inertia axes of the molecule. In this convention, Y is approximately aligned with the long axis of the pentacene backbone, X is normal to the pentacene skeleton, and Z lies approximately along the direction joining the two Si atoms.

S5. Experimental and calculated angular dependence of the high-frequency Raman response

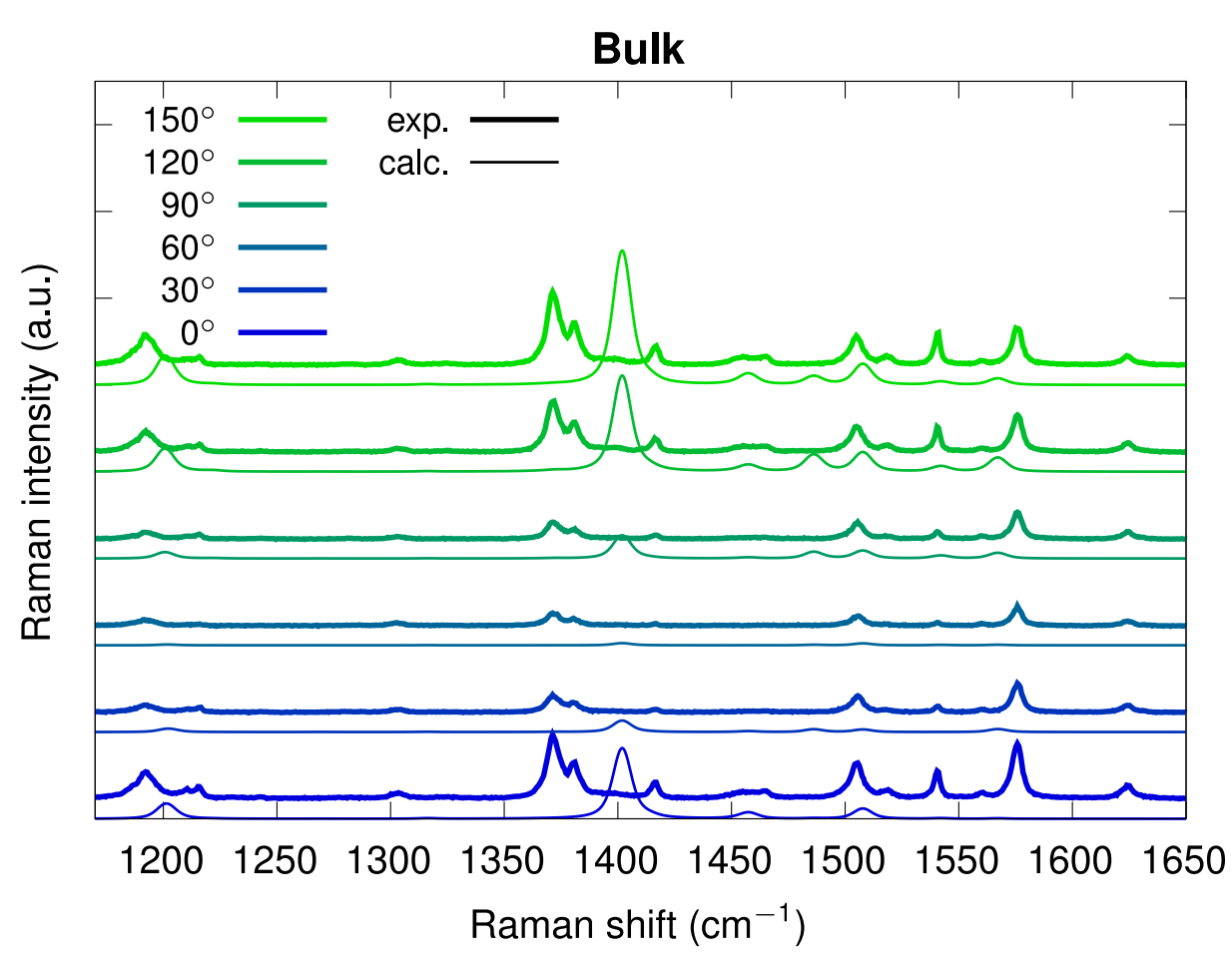

Figure S5. Experimental and calculated angular dependence of the high-frequency Raman response of TIPS-pentacene in the (ab) plane of the single crystal. The intensity modulation of the main intramolecular bands follows the same angular pattern in experiment and in the DFT simulations, supporting the assignment of the molecular-frame orientation used in the main text.

S6. Calculated low-frequency Raman fingerprints of TIPS-pentacene polymorphs

To place the structural assignment of the films on a broader basis, the calculated low-frequency Raman spectra of Forms I, II, and III were compared in the lattice-phonon region in Figure S6. Although partial overlap exists for some individual modes, the overall spectral profiles remain clearly different for the three polymorphs, showing that the low-frequency Raman response provides a robust fingerprint of the crystal form. Because the discussion in the main text concerns Raman-active vibrations, only Ag modes are listed in the tables below. For each mode, the frequency, the total rotational contribution R, and its decomposition along the three principal molecular inertia axes L, M, and N are reported.

Table S3. Calculated and experimental wavenumbers of the lowest-energy Raman-active modes of TIPS-pentacene Forms I, II, and III (with symmetry). The calculated squared rotational components around the three principal inertia axes L, M, and N are given, together with their sum; the axes are ordered according to increasing moment of inertia. Their correspondence with the molecular axes x,y,z shown in Figure S2 is also indicated.

FORM I						FORM II						FORM III					
Sym	Freq	R	R_L (R_z)	R_M (R_y)	R_N (R_{Lx})	Sym	Freq	R	R_L (R_z)	R_M (R_y)	R_N (R_{Lx})	Sym	Freq	R	R_L (R_z)	R_M (R_y)	R_N (R_{Lx})
Ag	28.9	58	4	54	0	Ag	28.3	73	3	0	69	Ag	20.5	56	2	0	54
Ag	35.8	69	2	1	66	Ag	42.6	71	0	68	2	Ag	40.8	76	1	74	1
Ag	46.0	31	8	6	17	Ag	49.1	44	22	16	6	Ag	56.1	2	2	1	0
Ag	52.5	10	4	1	5	Ag	58.5	15	0	1	14	Ag	60.4	34	0	9	25
Ag	53.7	11	11	0	0	Ag	60.3	5	0	2	2	Ag	64.2	26	13	4	9
Ag	72.7	31	0	31	0	Ag	75.6	3	1	0	2	Ag	73.3	9	1	7	1
Ag	77.0	25	24	0	1	Ag	98.8	1	0	0	1	Ag	90.8	6	1	2	4
Ag	84.8	0	0	0	0	Ag	109.1	6	0	5	1	Ag	101.5	3	1	0	2
Ag	90.0	6	0	1	5	Ag	124.8	1	1	0	0	Ag	108.8	2	0	0	2
Ag	93.2	8	0	5	3	Ag	127.4	8	7	0	0	Ag	121.8	1	0	0	0
Ag	98.6	7	6	0	1	Ag	141.3	2	1	0	1	Ag	137.7	7	6	1	0
Ag	109.0	2	1	0	1	Ag	147.5	9	4	3	2	Ag	140.3	5	3	1	1
Ag	121.9	3	3	0	0	Ag	172.8	1	0	0	0	Ag	163.8	9	8	0	0
Ag	128.5	34	34	0	0	Ag	181.1	8	8	0	0	Ag	179.6	28	27	0	1
Ag	142.8	0	0	0	0	Ag	187.2	7	6	0	0	Ag	192.6	2	1	0	0
Ag	172.2	1	1	0	0	Ag	197.4	31	30	1	0	Ag	201.6	5	5	0	0
Ag	179.2	0	0	0	0	Ag	219.3	0	0	0	0	Ag	217.2	4	4	0	0
Ag	188.5	0	0	0	0	Ag	224.8	11	11	0	0	Ag	222.7	12	12	0	0
Ag	206.0	0	0	0	0	Ag	243.7	1	1	0	0	Ag	236.9	10	10	0	0
Ag	213.6	0	0	0	0	Ag	246.9	1	1	0	0	Ag	245.6	0	0	0	0
Ag	224.2	0	0	0	0	Ag	263.2	1	1	0	0	Ag	257.1	0	0	0	0

Ag	227.4	0	0	0	0	Ag	274.0	0	0	0	0	Ag	271.6	1	1	0	0
Ag	233.5	0	0	0	0	Ag	282.0	1	0	0	0	Ag	275.9	0	0	0	0
Ag	237.0	0	0	0	0	Ag	299.2	0	0	0	0	Ag	288.8	0	0	0	0

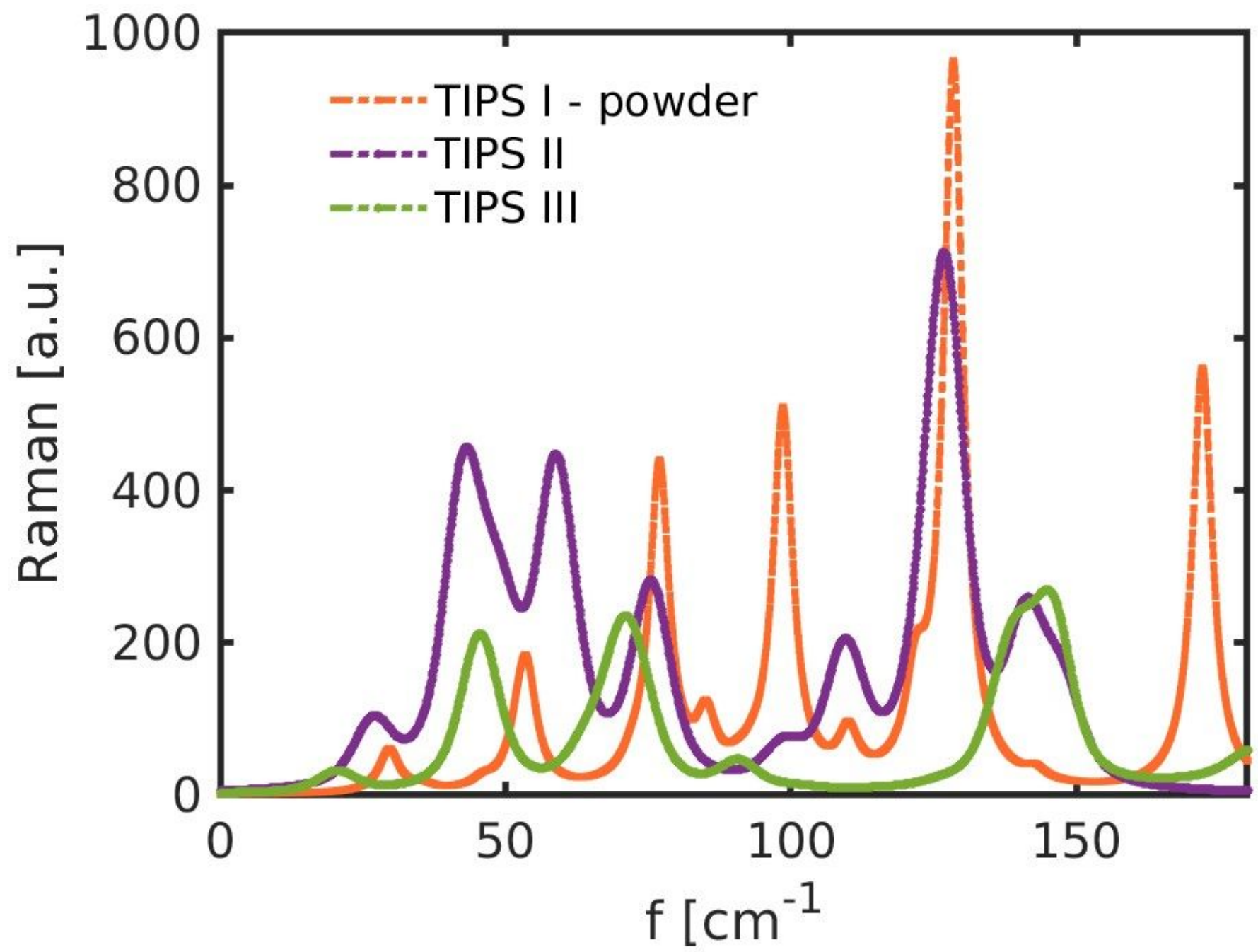

Figure S6. Calculated low-frequency Raman spectra of the reported TIPS-pentacene polymorphs. Despite partial similarities between some modes, the overall Raman fingerprints remain clearly polymorph dependent.

S7. Mode-resolved analysis of low-energy electron–phonon coupling

To clarify the microscopic origin of the EPC, the mode/q-point pairs giving the largest effective EPC/$\hbar\omega$ contributions were analyzed in terms of their molecular translational and rotational character, as reported in Table S4. In this representation, the phonon energy is explicitly taken into account, so that the reported quantity reflects not only the strength of the coupling itself but also the expected phonon population. Importantly, the largest effective contributions are associated with low-energy vibrations that retain substantial lattice character and, in several cases, marked rotational components involving the pentacene framework. These results show that, even in a Z = 1 crystal, low-energy optical modes, including modes with partial internal character, can contribute significantly to the modulation of the intermolecular electronic coupling. Although the present treatment does not retain the full q-

resolved EPC landscape, it includes the contribution of phonons beyond Γ through a branch-resolved average over the sampled Brillouin-zone points and is therefore consistent with the broader picture proposed for TIPS-pentacene, in which low-energy structural fluctuations strongly modulate intermolecular electronic coupling and play a key role in charge transport.

Table S4. The mode decomposition of the molecular translational and rotational character along the inertia axes of Table S3 for the mode/q-point pairs giving the highest effective EPC/ħω contributions; the phonon energy is used to account for the phonon population. As specified in Table S3, the L (z) axis connects the two TIPS-pentacene Si atoms, M (y) runs along the pentacene backbone, and N (x) is normal to the molecular plane, with increasing moment of inertia. Note that the three rigid translational degrees of freedom correspond to the acoustic branches at Γ. The analysis shows that, even in a Z = 1 crystal, internal vibrations may also contribute to the modulation of the electronic coupling and thus affect charge transport. The EPC contribution associated with each mode after DOS-weighted averaging over the full Brillouin zone is also reported.

q-point	Freq. [cm^{-1}] *Branch #*	EPC [meV/Å]	EPC$_{eff}$ [Å^{-1}]	sym.	T_L	T_M	T_N	R_L	R_M	R_N	mode EPC over the full BZ [meV/Å]
0.0 0.0 0.5	14.0 *# 1*	93.3	53.6	A	1	48	5	0	2	16	61.2
0.0 0.0 0.5	23.7 *# 4*	189.0	64.4	A	49	1	1	0	1	0	57.7
0.5 0.0 0.5	20.6 *# 1*	196.6	37.8	A	0	11	6	0	1	0	61.2
0.5 0.0 0.5	23.4 *# 2*	98.6	33.9	A	1	1	28	2	1	0	78.0
0.0 0.5 0.5	53.3 # 11	206.4	31.2	A	5	0	0	0	3	3	88.9
0.5 0.5 0.5	23.6 #1	84.0	28.7	A	10	3	2	1	1	3	61.2
